# Optimized DNA extraction and purification method for characterization of bacterial and fungal communities in lung tissue samples

**DOI:** 10.1038/s41598-020-74137-2

**Published:** 2020-10-15

**Authors:** Vicente Pérez-Brocal, Fabien Magne, Susana Ruiz-Ruiz, Carolina A. Ponce, Rebeca Bustamante, Viviana San Martin, Mireya Gutierrez, Gianna Gatti, Sergio L. Vargas, Andrés Moya

**Affiliations:** 1 Department of Genomics and Health, Foundation for the Promotion of Health and Biomedical Research of Valencia Region (FISABIO-Public Health), Avda. Cataluña 21, 46020 València, Spain; 2 CIBER in Epidemiology and Public Health (CIBEResp), Madrid, Spain; 3 Microbiology and Mycology Program, Biomedical Sciences Institute (ICBM), University of Chile School of Medicine, Av. Independencia 1027, Independencia, Santiago, Chile; 4 Médico Legal Institute of Chile, Santiago, Chile; 5 Institute for Integrative Systems Biology (I2SysBio), University of Valencia and Spanish National Research Council (CSIC), València, Spain

**Keywords:** Microbiology techniques, Microbial communities, Fungi

## Abstract

Human lungs harbor a scarce microbial community, requiring to develop methods to enhance the recovery of nucleic acids from bacteria and fungi, leading to a more efficient analysis of the lung tissue microbiota. Here we describe five extraction protocols including pre-treatment, bead-beating and/or Phenol:Chloroform:Isoamyl alcohol steps, applied to lung tissue samples from autopsied individuals. The resulting total DNA yield and quality, bacterial and fungal DNA amount and the microbial community structure were analyzed by qPCR and Illumina sequencing of bacterial 16S rRNA and fungal ITS genes. Bioinformatic modeling revealed that a large part of microbiome from lung tissue is composed of microbial contaminants, although our controls clustered separately from biological samples. After removal of contaminant sequences, the effects of extraction protocols on the microbiota were assessed. The major differences among samples could be attributed to inter-individual variations rather than DNA extraction protocols. However, inclusion of the bead-beater and Phenol:Chloroform:Isoamyl alcohol steps resulted in changes in the relative abundance of some bacterial/fungal taxa. Furthermore, inclusion of a pre-treatment step increased microbial DNA concentration but not diversity and it may contribute to eliminate DNA fragments from dead microorganisms in lung tissue samples, making the microbial profile closer to the actual one.

## Introduction

Although the lungs have traditionally been considered sterile, recent studies have revealed that the respiratory airways harbor a complex microbial community. While it is suspected that the airway microbiome is involved in colonization resistance of respiratory pathogens, and the development and integrity of the immune system, its role is not yet well understood^1^. Therefore, the lung-associated microbiome may provide new perspectives in understanding the pathogenesis of airway infections and chronic lung diseases. However, its analysis involves technical challenges for researchers. Like all culture-independent techniques, data quality depends on the effectiveness of extracting microbial DNA. Different DNA extraction methods generate different microbial profiles in the same fecal samples due to cell wall disruption^2–4^. Additionally, as the lung microbiome contains a low biomass, the microbial DNA extracted can be insufficient for detection by polymerase chain reaction (PCR). Moreover, high concentration of human genomic DNA (gDNA) compared to microbial DNA^5^ can inhibit the PCR. Thus, it is crucial to develop an efficient method of extracting DNA from lung microorganisms. These factors may distort the apparent composition of microbial communities based on PCR, such as high-throughput sequencin^6,7^.

Samples with low microbial biomass are more sensitive to DNA contaminants introduced during sample processing from laboratory reagents, extraction kits or the laboratory environment^8–11^. Although microbiome studies analyze low microbial biomass samples, they routinely do not analyze negative controls from DNA extractions and/or sequencing; even reporting statistically noteworthy taxa that overlap those observed in negative controls^10,12,13^. Thus, microbial contaminants in low microbial biomass samples, such as lung tissue, can alter the relative abundance of the microbial communities under analysis; however, this is often overlooked.

Many investigations on the airway microbiome use DNA extraction methods that have not been previously validated. Thus, here we present validation of a new protocol for DNA extraction and microbiome analysis in lung tissue samples. This study compares five protocols for assessing bacterial and fungal DNA recovery, subsequent microbial PCR detection, and the resulting bacterial and fungal community structures in order to define the optimal extraction method for use in lung microbiome studies.

## Results

### Quality and quantity of genomic DNA from five extraction protocols

**Figure 1:**
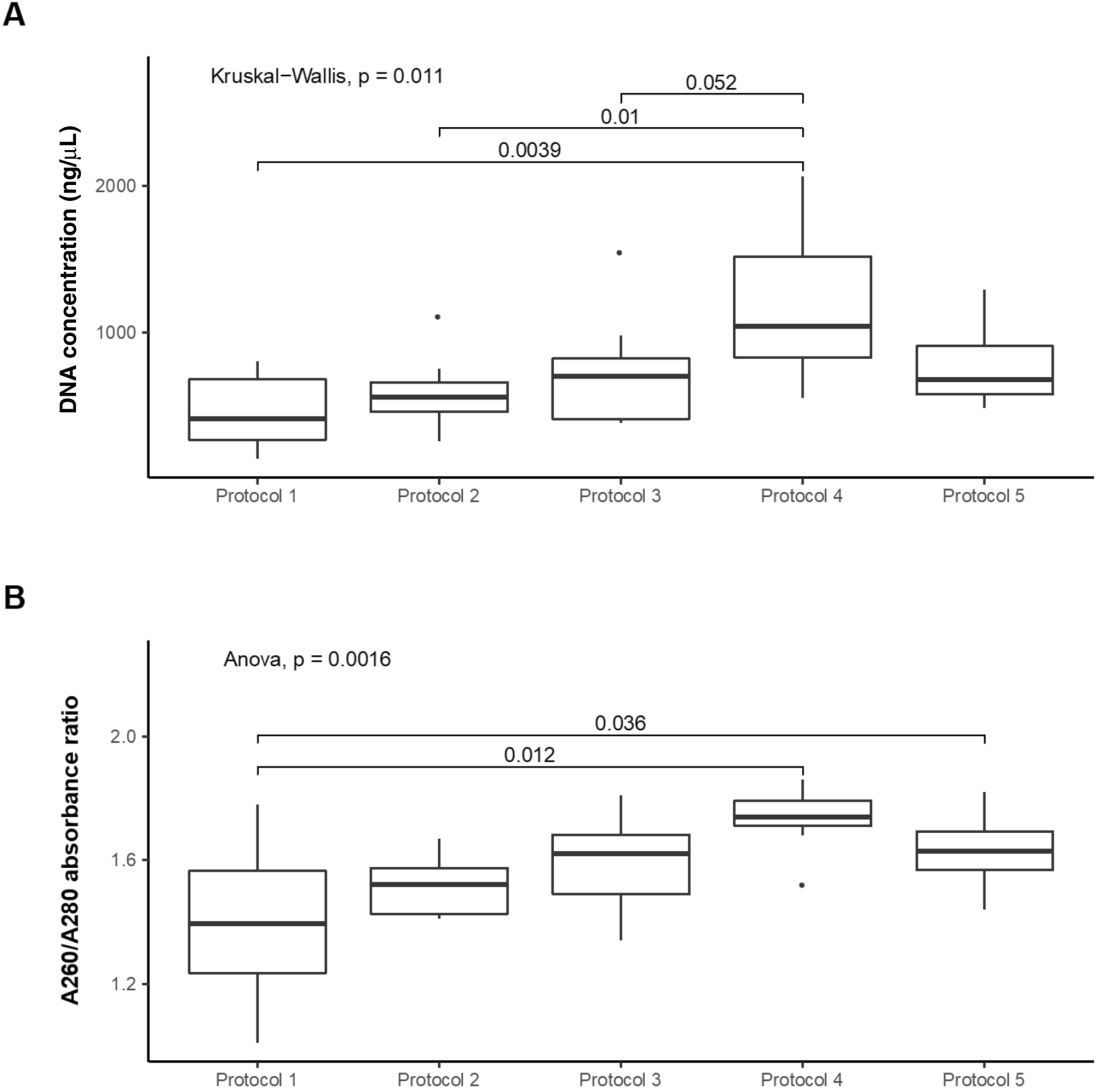
Comparison of the yield and purity of extracted DNA between protocols. (**A**) DNA yield and (**B**) DNA quality (n = 7 samples/protocol). DNA yield is expressed as DNA concentration (ng/μl) normalized by quantity of lung tissue used for DNA extraction. Solid black lines indicate the median, and the lower and upper bounds of the box represent the 25 and 75% quartiles. Outliers, defined as falling outside the 10% and 90% quartiles, are indicated with black circles. Significant differences specified in the figure are based on ANOVA or the Kruskal–Wallis tests.

Lung tissue specimens from seven autopsied individuals were processed for DNA extraction following the five methods described (Table 1) and the concentration and quality measured (Fig. 1A). Protocol 2 including the bead-beating and 3 including the Phenol:Chloroform:Isoamyl alcohol step, had slightly increased DNA yields compared to protocol 1; however these findings were not significant. Combining both additional steps, protocol 4 significantly increased DNA concentrations (1,104.8 ± 522.7 ng/µl) compared to protocols 1 (525.4 ± 460.2 ng/µl, p = 0.003) and 2 (557.4 ± 245.2 ng/µl, p = 0.036). Protocol 5 (686.2 ± 172.3 ng/µl) also showed a relative, but non-significant, increase in DNA compared to protocol 1.

**Table 1:** DNA extraction protocols used in this study.

Protocol	Pre-treatment step	Phenol:Chloroform:Isoamyl alcohol step	Bead-beating step	QIAamp DNA Mini kit (QIAGEN)
1	−	−	−	+
2	−	−	+	+
3	−	+	−	+
4	−	+	+	+
5	+	+	+	+

As for DNA quality, protocol 1 had the lowest 260/280-absorbance ratio (mean ± SD, 1.39 ± 0.25) compared to other protocols (Fig. 1B), being the highest in protocols 3 (1.59 ± 0.16, p = 0.090), 4 (1.73 ± 0.10, p = 0.012) and 5 (1.63 ± 0.12, p = 0.036), which included a Phenol:Chloroform:Isoamyl alcohol step.

### Evaluation of human, bacterial, and fungal DNA in the five extraction protocols

**Figure 2:**
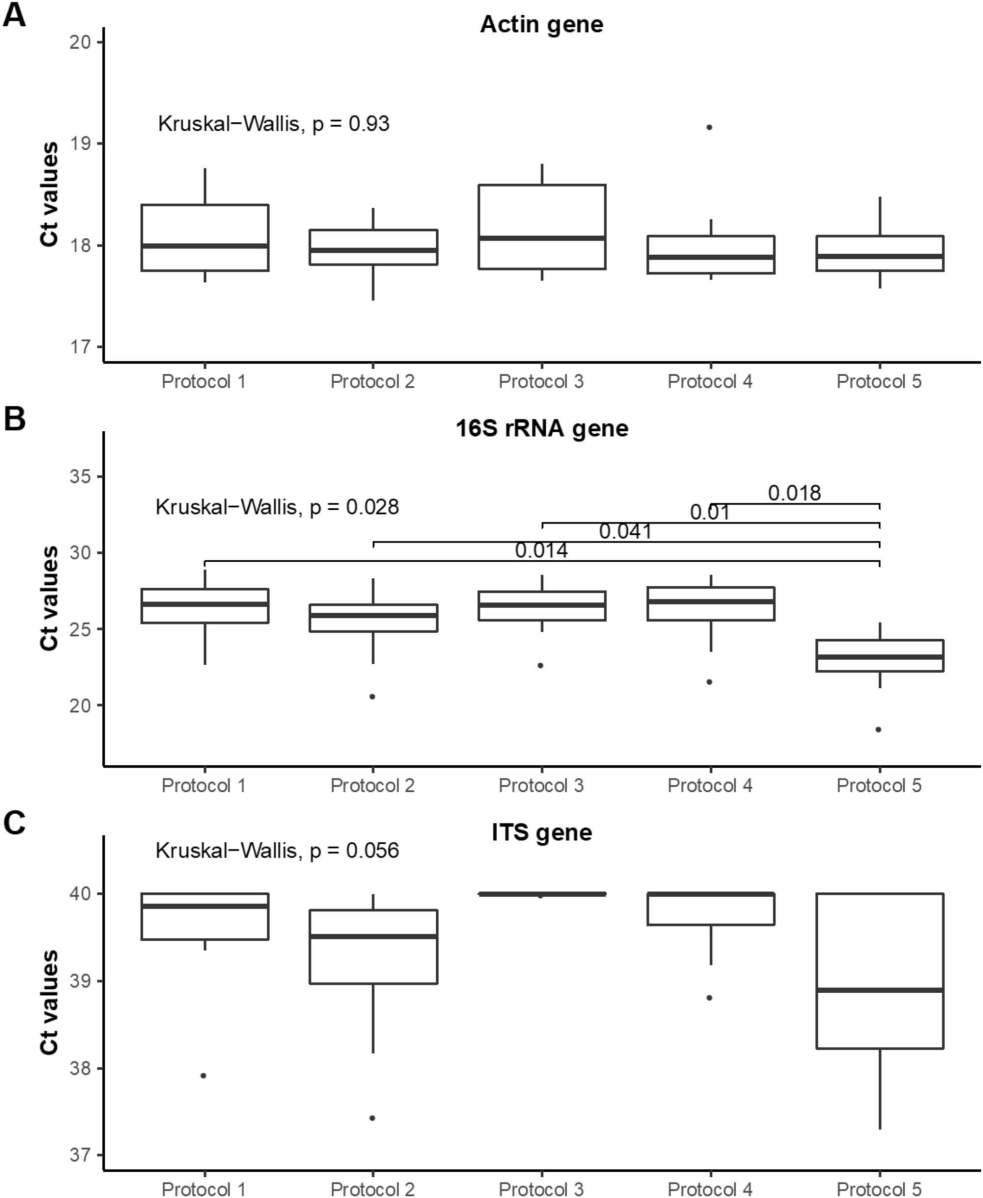
Evaluation of human, bacterial and fungal DNA by extraction protocol. Boxplot of Ct values for β-actin gene (**A**), 16S rRNA (**B**) and 18S rRNA gene (**C**) obtained with the five extraction protocols. Solid black lines indicate medians, and the lower and upper bounds of the box represent the 25 and 75% quartiles. Outliers, defined as falling outside the 10% and 90% quartiles, are indicated with black circles. Significant differences based on the Kruskal–Wallis test are specified in the figure.

The evaluation of the content of human, bacterial, and fungal genomes through qPCR amplification of human β-actin, 16S rRNA and 18S rRNA genes respectively showed that the DNA extraction method did not affect the *C*t obtained for the β-actin gene, suggesting that the level of human DNA was equivalent for all extraction protocols (Fig. 2A). However, the *C*t of the 16S rRNA gene (mean ± SD) was significantly decreased with protocol 5 (22.9 ± 2.2) compared to all others (26.3 ± 2.2 for protocol 1; 25.3 ± 2.5 for protocol 2; 26.3 ± 1.9 for protocol 3; and 26.1 ± 2.4 for protocol 4) (Fig. 2B).

We detected low quantities of fungi in lung tissue, as the *C*t values of 18S RNA gene ranged from 37.3 to 40 (Fig. 2C). Although there were no significant differences, the *C*t tended to be decreased in samples processed with protocol 5 (38.9 ± 1.0) compared to others (39.6 ± 0.7 for protocol 1; 39.2 ± 0.9 for protocol 2; 40.0 ± 0.0 for protocol 3; and 39.7 ± 0.5 for protocol 4).

### Impact of bacterial and fungal contamination of DNA

**Figure 3:**
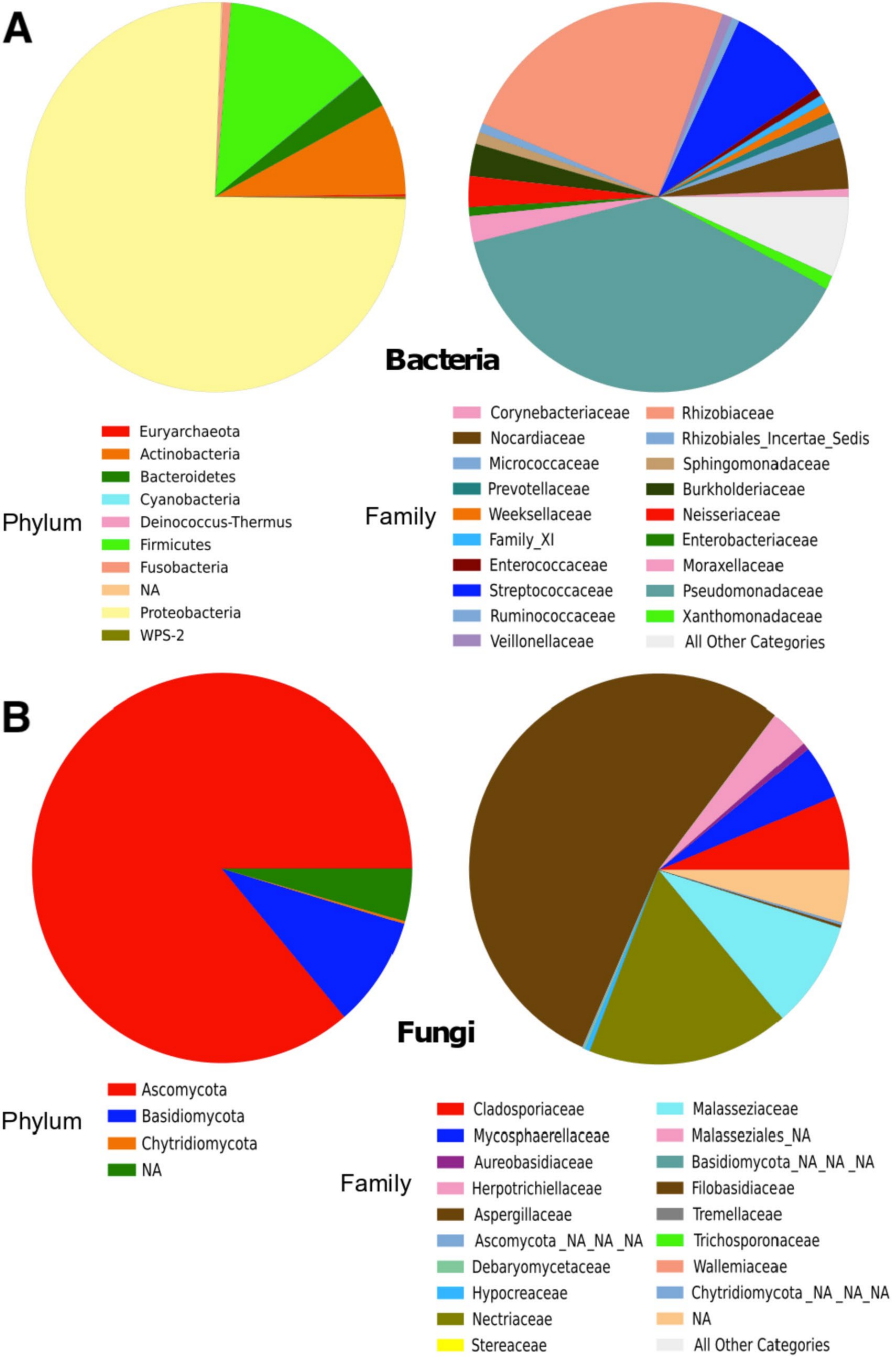
Relative abundance of phyla and families identified in negative controls during the DNA extraction process. Sequencing of the 16S rRNA gene (**A**) and the ITS region (**B**) carried out on four negative controls using the Illumina MiSeq platform. A complete list of taxa is provided in the Supplementary Table S1.

Sequencing of the 16S rRNA and ITS genes of four negative controls yielded (mean ± SD, 5689 ± 3268 bacterial sequences per sample (22,756 reads in total) and 18,259 ± 5228 fungal sequences per sample (73,037 reads in total). In comparison, sequencing of samples generated average 28,737 bacterial reads and 74,863 reads per sample. Overall, we detected 55 bacterial families belonging to 8 phyla in negative controls, represented by Proteobacteria (75.3%), Firmicutes (12.9%), and Actinobacteria (7.6%), followed by Bacteroidetes, Fusobacteria, Deinococcus-Thermus, and others (< 3% each) (Fig. 3A). We also detected an archaeal phylum Euryarchaeota (0.19%). About 80% of the reads were assigned as Pseudomonaceae (38.5%), Rhizobiaceae (24.2%), Streptococcaceae (8.6%) or Nocardiaceae (4.3%) (Fig. 3A and Supplementary Table S1). For fungi (Fig. 3B), we identified 13 families belonging to 3 phyla: Ascomycota (86.2%), Basidiomycota (9.2%) and Chytridiomycota (0.23%). Nearly 9 out of every 10 reads corresponded to Aspergillaceae (53.9%), Nectriaceae (17.1%), Malasseziaceae (9.0%) or Cladosporiaceae (6.2%) (Supplementary Table S1).

To evaluate the contamination level of our samples, we identified reads mapping 100% identity with those of negative controls. For bacteria, the number of reads removed was 337,935 (61.7% of the reads before clustering), reaching 9655 ± 13,707 (mean ± SD) reads per sample. For fungi, figures were lower, with 168,683 (8.7% of the reads) and 4820 ± 7295 reads per sample. The negative controls contained 1580 reads (6.9%) showing 100% identity with at least one bacterial sample, whereas in fungi this figure was lower, 258 reads (0.4%). Those reads were removed from the samples. Despite the relatively high number of reads in the negative controls, most of them were actually unique and restricted, as a consequence the impact of the removal of reads due to potential contamination on the samples was relatively low. To ensure that the microorganisms detected in our samples were not completely a result of contamination, we compared the microbial community of our samples with that of negative controls by performing a CCA based on Bray–Curtis distances for bacterial and fungal communities (Supplementary Fig. S1A,B). We observed that the majority of samples and negative controls clustered separately for both bacteria and fungi, which was confirmed by the Adonis test (p = 0.003 for bacteria and p = 0.017 for fungi).

### Lung microbial community diversity

Sequencing of the V3–V4 regions of the 16S rRNA gene carried out on the 35 samples from the 7 individuals using 5 protocols yielded 1,005,778 raw reads, averaging 28,737 reads per sample, and ranging from 4717 to 137,755 reads. Similarly, the sequencing of the ITS regions in these samples, resulted in 2,620,221 raw reads with a mean read count of 74,863 reads per sample, ranging from 18,150 to 256,441 reads.

After sequence filtering, merging, chimera, and host reads removal, there were 547,468 reads (mean 15,642, ranging from 2632 to 88,859 reads) for bacteria, and 1,945,654 reads (mean 55,590, ranging from 10,685 to 201,390 reads) for fungi.

**Figure 4:**
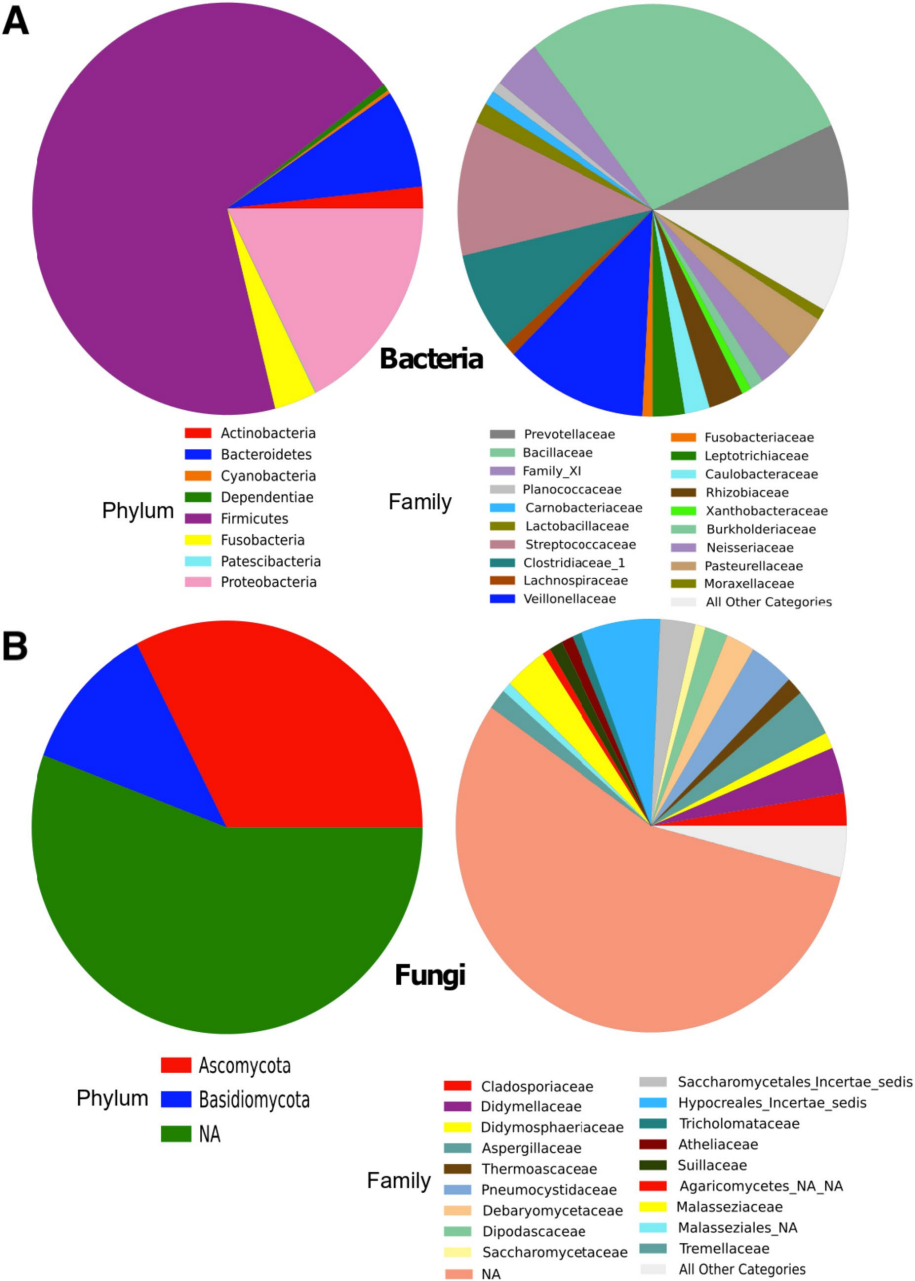
Relative abundance of phyla and families identified in lung tissue samples. Sequencing of the 16S rRNA gene (**A**) and the ITS region (**B**) carried out on 7 lung tissue samples using the Illumina MiSeq platform. A complete list of taxa is provided in Supplementary Table S2.

After removal of sequences that clustered with negative control reads at 100% identity the composition and abundance of the remaining 209,533 bacterial and 1,776,971 fungal “contaminant-free” reads are summarized in Fig. 4 (see Supplementary Fig. S2 for individual data).

For bacteria (Fig. 4A), 54 families were identified, mainly from phyla Firmicutes (68.7%), Proteobacteria (17.5%), Bacteroidetes (7.7%), Fusobacteria (3.5%) and Actinobacteria (1.70%). The three most abundant families, Bacillaceae (28.7%), Veillonelaceae (11.8%), and Streptococcaceae (10.6%), accounted for half of the reads. For a complete record of bacterial families, see Supplementary Table S2.

In the case of fungi (Fig. 4B), we identified 38 families, mainly from phyla Ascomycota (32.6%) and Basidiomycota (11.7%); however, most reads could not be assigned (55.7%). Unlike bacteria, no dominant families were identified for fungi, with the most abundant ones Hypocreales_fam_Incertae_sedis (6.6%), Aspergillaceae and Pneumocystidaceae (3.7% each), Didymellaceae (3.6%) and Malasseziaceae (3.5%). These families accounted for roughly 2 of every 10 reads. For a complete record of fungal families identified see Supplementary Table S2.

### Impact of DNA extraction protocol on the bacterial community

**Figure 5:**
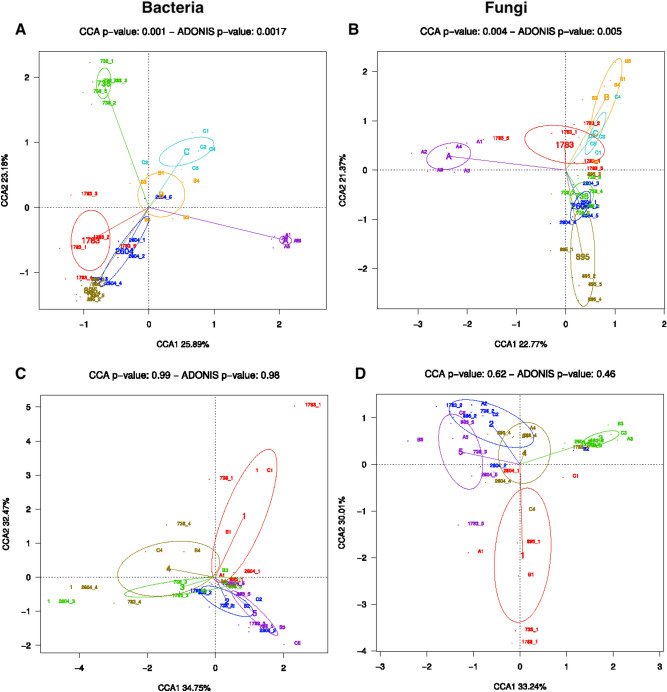
Canonical correspondence analysis (CCA) plots of bacterial and fungal microbiomes according to individuals (**A**, **B**, respectively) and according to the DNA extraction protocol (**C**, **D**, respectively).

We investigated the effect of the extraction protocols on bacterial community detection in lung tissue samples. In the Fig. 5, CCA analysis shows that DNA extraction protocols did not drive significant differences. Rather, samples clustered by individual rather than by extraction protocol (Fig. 5). Furthermore, there were no significant differences in the Shannon index between the different DNA extraction protocols (Supplementary Fig. S3).

**Figure 6:**
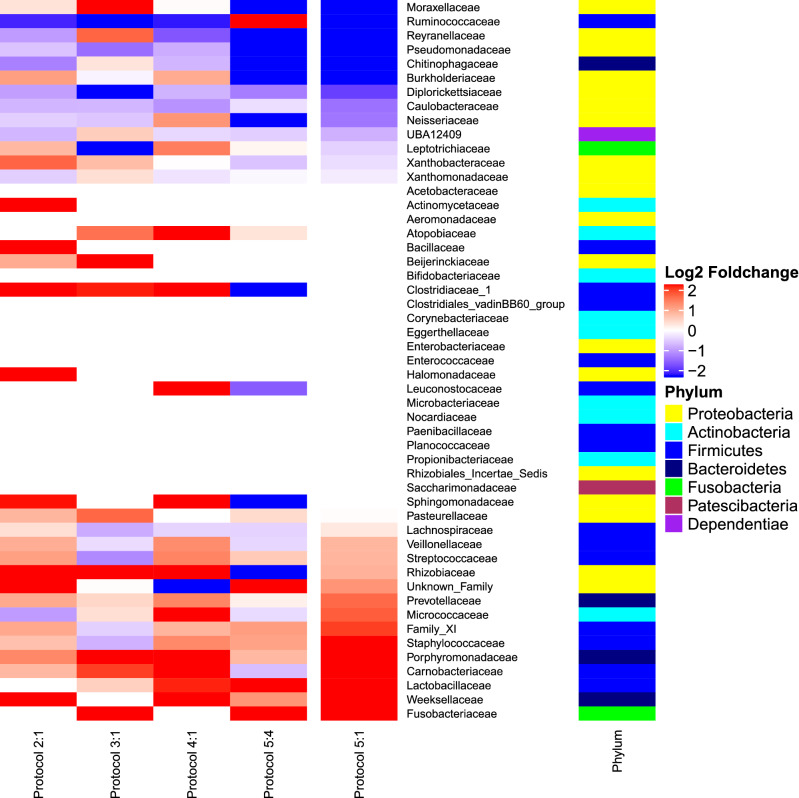
Bacterial changes associated with bead beating (comparison of protocol 2 with 1); the Phenol:Chloroform:Isoamyl alcohol step (comparison of protocol 3 with 1); bead-beating and the Phenol:Chloroform:Isoamyl alcohol steps (comparison of protocol 4 with 1); pre-treatment steps (comparison of protocol 5 with 4); and pre-treatment, the bead-beating and the Phenol:Chloroform:Isoamyl alcohol steps (comparison of protocol 5 with 1). Average of log_2_ fold-change in relative taxon abundance was calculated using the DESEQ2 software.

Nevertheless, we observed that variations in the DNA extraction protocols (Fig. 6) did drive changes in the relative abundance of some taxa (see Supplementary Fig. S4 and Table S3 for individual data). The DESEQ2 software used to analyze family-level fold changes in taxa between the distinct protocols showed that the addition of a bead-beating step (protocols 2 and 4) improved the detection of families (40 and 37 detected with protocols 2 and 4 respectively) compared to protocol 1 (34). Based on log_2_ transformed relative abundance, twenty-two taxa were increased in protocol 2 compared to protocol 1. We observed increases, log_2_ fold-change > 1, in families belonging to the phylum Firmicutes, such as Bacillaceae (+ 26.2), Clostridiaceae_1 (+ 3.5) and Streptococcaceae (+ 1.0). Also, in the phylum Bacteroidetes families Weeksellaceae (+ 21.3) and Porphyromonadaceae (+ 1.2) were increased in protocol 2; in the phylum Actinobacteria the family Actinomycetaceae (+ 7.3) was increased. Additionally, we observed increases in the phylum Proteobacteria, including families Halomonadaceae (+ 21.8), Rhizobiaceae (+ 4.8), Unknown_Family (+ 2.3), Sphingomonadaceae (+ 2.0), Xanthobacteraceae (+ 1.5) and Burkholderiaceae (+ 1.0).

The Phenol:Chloroform:Isoamyl alcohol step also affected the detection of some family taxa (34 families detected), although to lesser extent than bead beating. Comparisons between protocols 3 and 1 showed that the Phenol:Chloroform:Isoamyl alcohol step improved the detection of 18 families of the phylum Fusobacteria, including Fusobacteriaceae (+ 23.7); the phylum Bacteroidetes, including Porphyromonadaceae (+ 2.3); the phylum Actinobacteria, including Atopobiaceae (+ 1.4); the phylum Firmicutes, including Clostridiaceae_1 (+ 2.0) and Carnobacteriaceae (+ 1.8); and the phylum Proteobacteria, including Beijerinckiaceae (+ 4.4), Rhizobiaceae (+ 4.4), Moraxellaceae (+ 2.2), Reyranellaceae (+ 1.5) and Pasteurellaceae (+ 1.5).

The combination of the bead-beating and Phenol:Chloroform:Isoamyl alcohol steps (protocol 4) appeared to have a synergistic effect on the abundance of bacterial taxa (37 families detected). Comparisons of the relative abundance observed in protocols 4 and 1 revealed increased log_2_ fold changes in 20 bacterial families; these were the primarily the same families (18/20) that were increased in protocols 2 and/or 3. Increase family-level taxa, log_2_ fold-change > 1, belonged to the phylum Actinobacteria, including Atopobiaceae (+ 5.9) and Micrococcaceae (+ 2.7); the phylum Bacteroidetes, including Weeksellaceae (+ 2.8), Porphyromonadaceae (+ 2.1) and Prevotellaceae (+ 1.2); the phylum Firmicutes, including Clostridiaceae_1 (+ 3.6), Carnobacteriaceae (+ 3.3), Leuconostocaceae (+ 2.0), Lactobacillaceae (+ 1.9), Streptococcaceae (+ 1.2), Staphylococcaceae (+ 1.2) and Veillonellaceae (+ 1.2); the phylum Fusobacteria, such as Leptotrichiaceae (+ 1.3); and the phylum Proteobacteria, such as Rhizobiaceae (+ 3.4), Sphingomonadaceae (+ 2.9) and Neisseriaceae (+ 1.1).

Fewer families, 31 were identified in protocol 5; however, the pre-treatment step did increase the detection of some of them. Comparisons between protocol 4 and 5 showed an average log_2_ fold-change increase (> 1) in 13 families, belonging to the phylum Firmicutes, including Lactobacillaceae (+ 3.2), Ruminococcaceae (+ 3.0), Family_XI (+ 1.0) and Staphylococcaceae (+ 1.0); the phylum Bacteroidetes, including Weeksellaceae (+ 1.1); the phylum Proteobacteria, including Unknown_Family (+ 6.1); and the phylum Fusobacteria, including Fusobacteriaceae (+ 6.1).

As compared to protocol 1, protocol 5 increased detection for 15 families. Among those, we observed an increased family abundance, log_2_ fold-change > 1, in the phylum Firmicutes, including Lactobacillaceae (+ 3.5), Carnobacteriaceae (+ 2,8), Staphylococcaceae (+ 2.3) and Family_XI (+ 1,8); the phylum Actinobacteria, including Micrococcaceae (+ 1.6); the phylum Bacteroidetes, including Weeksellaceae (+ 4.3), Porphyromonadaceae (+ 2.5) and Prevotellaceae (+ 1.5); the phylum Fusobacteria, including Fusobacteriaceae (+ 6,2); and the phylum Proteobacteria, including Unknown_Family (+ 1.1).

In contrast, we observed that 13 family taxa were decreased in protocol 5 compared to protocol 1, log_2_ fold-change < 0, primarily belonging to the phylum Proteobacteria (9/13), as well as the phyla Firmicutes (1/13), Bacteroidetes (1/13), Dependentiae (1/13), and Fusobacteria (1/13). Most of these families (11/13) were also decreased in protocols 2, 3 and/or 4.

### Impact of DNA extraction protocol on the fungal community

We investigated whether the DNA extraction protocols affected the composition of fungal community. CCA analysis showed that samples tend to cluster by extraction protocol, however this grouping was not statistically significant, suggesting that the extraction method affects the fungal community (Fig. 5). In addition, we observed that samples cluster according to the subjects (Fig. 5). Furthermore, there were no significant differences in the Shannon index between the different DNA extraction protocols (Supplementary Fig. S3).

Compositional comparison revealed that the fungal families identified ranged from 9 in protocol 5 to 24 in protocol 2, with figures for the other protocols of 13 (protocol 4), 15 (protocol 3), and 17 (protocol 1). We noted that the bead-beating step improved fungal family retrieval (protocol 2), although this increase was not found for protocol 4, which also the Phenol:Chloroform:Isoamyl alcohol steps.

**Figure 7:**
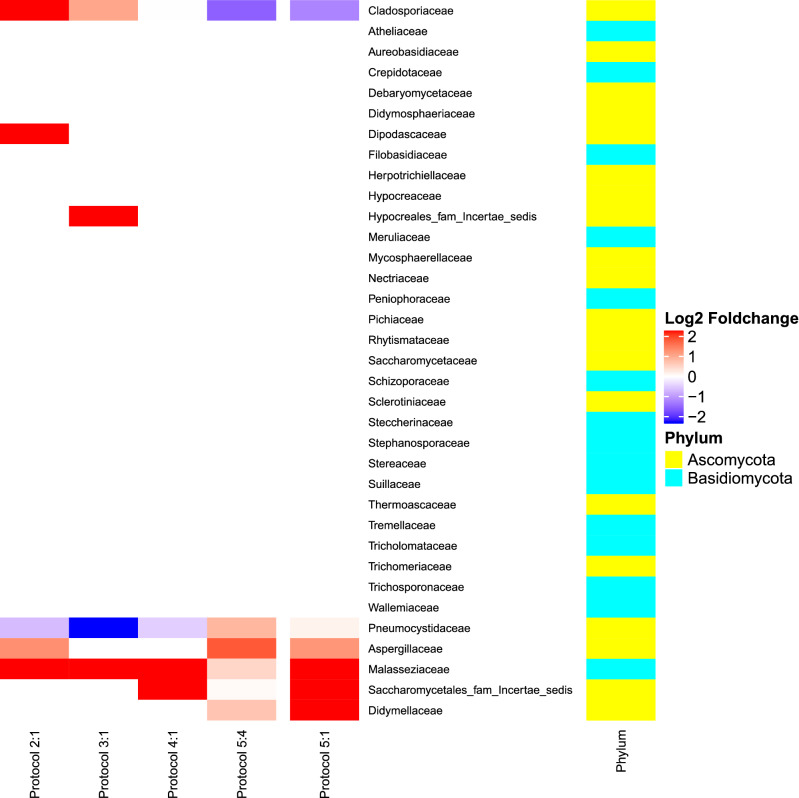
Fungal changes associated with bead beating (comparison of protocol 2 with 1); the Phenol:Chloroform:Isoamyl alcohol step (comparison of protocol 3 with 1); the bead-beating and the Phenol:Chloroform:Isoamyl alcohol steps (comparison of protocol 4 with 1), the pre-treatment steps (comparison of protocol 5 with 4); and the pre-treatment, the bead-beating and the Phenol:Chloroform:Isoamyl alcohol steps (comparison of protocol 5 with 1). Average of log_2_ fold-change in relative taxon abundance was calculated using the DESEQ2 software.

To determine the effect of bead beating, we compared changes in the relative abundance of taxa between protocols 2 and 1. The following families showed log_2_ fold-change increases in protocol 2 (Fig. 7): Malasseziaceae (+ 6.2), Cladosporiaceae (+ 3.5), Dipodascaceae (+ 2.6) and Aspergillaceae (+ 1.1) (see Supplementary Fig. S5 and Table S3 for individual data).

In contrast, the Phenol:Chloroform:Isoamyl alcohol step appeared to induce fewer changes (protocol 3) compared to protocol 1, as similar number of families were recovered (15 and 17 detected, respectively). However, log2 fold-change increases were observed in the phylum Basidiomycota, including the family Malasseziaceae (+ 5.8) and in the phylum Ascomycota, including Hypocreales_fam_Incertae_sedis (+ 5.2) and Cladosporiaceae (+ 0.9).

We observed, associated to the combination of the bead-beating and Phenol:Chloroform:Isoamyl alcohol steps, protocol 4, compared to protocol 1 increases in the log2 fold-change in the family Malasseziaceae (+ 6.2), which was also increased protocols 2 and 3. In addition, the family Saccharomycetales_fam_Incertae_sedis (+ 28.3) was also increased.

Although protocol 5 recovered fewer families than protocol 1 (9 versus 15, respectively), comparison between protocols 5 and 4 revealed that the pre-treatment step seems to improve retrieval of the families Aspergillaceae (log_2_ fold-change + 1.6), Pneumocystidaceae (+ 0.8), Didymellaceae (+ 0.6) and Saccharomycetales_fam_Incertae_sedis (+ 0.1) from the phylum Ascomycota; and Malasseziaceae (+ 0.4) from the phylum Basidiomycota.

All family taxa increased in protocol 5 compared to protocol 1 were also increased by the bead-beating, the Phenol:Chloroform:Isoamyl alcohol or the pre-treatment steps: Didymellaceae (log_2_ fold-change + 29.3), Saccharomycetales_fam_Incertae_sedis (+ 28.9), Aspergillaceae (+ 1.1) and Pneumocystidaceae (+ 0.1) from the phylum Ascomycota; and Malasseziaceae (+ 7.2) from the phylum Basidiomycota.

## Discussion

Here, using near-death autopsied lungs from people dying in accidents, we provide the first description of the bacterial and fungal community inhabiting the lungs in healthy human subjects. Furthermore, we evaluated the effect of five different DNA extraction protocols on the quantity and quality of nucleic acids, as well as on the bacterial and fungal community composition. We show that additional steps, including bead-beating, Phenol:Chloroform:Isoamyl alcohol, and pre-treatment affect the characterization of the microbial community in lung tissue samples.

Over the last decade, the development of culture-independent techniques for microbiological analysis has uncovered the existence of a microbial community in the lung, which was previously considered to be sterile. The lower respiratory tract of healthy subjects was mainly colonized by anaerobic bacteria, such as the phylum Bacteroidetes including the family Prevotellaceae and the phylum Firmicutes including the families Veillonellaceae and Streptococcaceae, as well as aerobic bacteria, such as the phylum Proteobacteria including the families Pseudomonaceae, Neisseriaceae, and Pasteurellaceae^14,15^. However, these data are based on studies whose samples were contaminated by microorganisms of the upper respiratory tract (i.e. sputum, bronchial aspirate, lung biopsy and bronchoalveolar lavage), as during their collection they transited through the oropharynx; thus, they do not represent the commensal communities inhabiting the lung^16^. Except for sputum, the collection of these samples is ethically difficult to perform in healthy subjects, as they require invasive procedures, making the characterization of the healthy lung microbiome extremely difficult.

In this study, we choose to use lung tissue samples obtained near death from subjects for characterizing the lung microbiome, as the initial postmortem microbiome would be a reflection of the host microbiome preceding death. The microbiome composition of distinct body habitats during the first 48 h postmortem showed still differences and remained stable in the first two days after death compared to those analyzed at a later time^17^. These data suggest that anatomic locations were not still contaminated by postmortem transmigration. In addition, the lung, like the brain, would be less sensitive to postmortem biological changes, compared to other organs. In effect it was shown that the lung was less sensitive to postmortem mRNA degradation^18,19^. As the microcirculation continues working for some time after death, it is hypothesized that tissues with abundant small blood vessels and microcirculation such as brain and lung would be less sensitive to postmortem changes. Therefore, despite the fluidic process of decomposition, the body can maintain its microbial communities with strong niche differentiation among anatomic locations. Together these data highlight the valuable biological resource of the human postmortem tissue (within 48 h of death) for characterizing the lung microbiome.

Our study shows that lung tissue samples from healthy subjects were composed mainly of the phylum Firmicutes, largely represented by the families Anaerobacillaceae, Streptococcaceae, Clostridiaceae and Veillonellaceae. We also detected the phyla Bacteroidetes, mainly represented by the family Prevotellaceae, and Proteobacteria, mainly the families Neisseriaceae and Pasteurellaceae, in lower concentrations. We observed great diversity in the microbiome inhabiting the lung. Although many fungal sequences remain unidentified, due to a lack of reference genomes^20^, the families we identified came primarily from the phyla Ascomycota and Basidiomycota, as well as Saccharomycetae, Saccharomycetales family Incertae sedis, and Aspergillaceae. Interestingly, species of some identified families such as *Candida*, *Malassezia* and *Saccharomyces*, have been previously reported to cause lung infection, suggesting that they may be present in the lungs as commensals^21^.

The study of the lung microbiota remains a technical challenge for researchers due to the low microbial biomass inhabiting the human lung, estimated at approximately 2.2 × 10^3^ bacterial genomes per cm^2^^14,22^. Consequently, these samples are more sensitive to contamination during the DNA extraction process, affecting sequencing data and interpretation of the microbiota. This is even more of a concern for fungi, present in lower concentrations in our samples compared to bacteria. This problem was well illustrated in a study by Lauder and collaborators, who characterized the microbiota of the placenta, which is also composed of a low microbial biomass^23^. The authors were unable to detect significant differences between the placental samples and contamination controls. In contrast, our study shows a significant separation between lung tissue samples and environmental contamination.

However, we identified potential contaminants in our samples with bacterial contamination more common than fungal one (67.7% vs 8.7% of the reads). Contaminants were identified through a computational approach, based on 100% identity sequences present in both negative controls and samples. This strategy appears more successful in removing sequences, which is vital as the lung microbiota can be contaminated by the bacterial content of inhaled air. It is likely that some bacteria of the lung microbiota are the same found in contaminant indoor air. Nevertheless, it is probable that some taxa from the lung microbiota were removed through our strategy. As the length of sequencing reads was only about 460 bp, distinct microbial strains present in the lung microbiota and in the contaminants could have 100% identity sequences. Thus, we probably overestimated the level of contamination in our samples.

In microbiome studies, it is well known that DNA extraction protocols are directly related to the quality of sequencing and taxonomic identification of microorganisms. The need for high concentration and quality of extracted DNA is of special concern in low biomass samples, such as lung tissue samples. In addition to the low microbial concentration, lung tissue contains mostly human DNA, making it harder to amplify and sequence microbial DNA. In this context, choosing an adequate DNA extraction protocol ensures an efficient recovery of all microorganisms present in the samples and the quality of the extracted DNA.

In our study, the addition of a bead-beating step in protocol 2 did not significantly increase the recovery of extracted DNA compared with protocol 1. However, the mechanical action of the beads on the microbial wall improves microbial disruption favoring the detection of Gram-positive bacteria from the phyla Firmicutes (e.g. families Bacillaceae and Clostridiaceae) and Actinobacteria (e.g. Actinomycetaceae and Atopobiaceae) that have many layers of peptidoglycan in their thick cell wall, which is not easily disrupted. Moreover, although the Proteobacteria were reduced, we also retrieved increased abundances in some Gram-negative bacteria from the phyla Bacteroidetes (e.g. families Weeksellaceae and Porphyromonadaceae) and Proteobacteria (e.g. Halomonadaceae, Rhizobiaceae and Sphingomonadaceae). Previous DNA extraction methods based on enzymatic treatment without physical disruption showed reduced recovery of Gram-positive bacteria and relatively elevated levels of Gram negatives^24,25^. It is noteworthy that fungi often have a cell wall that is harder to lyse than bacterial cell walls, and DNA extraction kits are generally not optimized for fungal DNA extractions. We observed a higher abundance of the families Malasseziaceae and Aspergillaceae in samples processed with bead beating. It has been previously shown that extraction protocols that employ bead beating increase DNA yields of genera from the Aspergillaceae^26^ and the Malasseziaceae families^27^. In addition to these two families, our study revealed that the bead-beating step also increased the recovery of other fungal taxa, such as the families Cladosporiaceae and Dipodascaceae.

On the other hand, the Phenol:Chloroform:Isoamyl alcohol step alone in protocol 3 leads to modest changes in the bacterial and fungal communities. However, we observed that this step ensures a better quality of the extracted DNA (ratio 260/280-absorbance) compared to protocol 1. While the phenol can aid in disrupting the cell wall by denaturing protein and lipids, it also permits the removal of PCR inhibitors by separating nucleic acids from other compounds.

When the bead-beating and phenol–chloroform steps were combined (protocol 4), they resulted in the highest total DNA concentration and the best quality of extracted DNA (Fig. 1). Nevertheless, the bacterial and fungal DNA, estimated by qPCR, was not significantly elevated compared to protocol 1. Moreover, the majority of bacterial and fungal taxa increased in protocol 4 were also increased in protocol 2 and/or the protocol 3. Subsequently, we added a pre-treatment step using PBS 1× under agitation and filtering on gauze and centrifugation to harvest microbes. Compared to protocol 1, we did not observe a loss of total DNA in lung tissue samples processed with the protocol 5. In contrast, higher concentrations of bacterial and fungal DNA, determined by qPCR, were observed compared to the remaining protocols. These results were expected, as the filtering and centrifugation steps in pre-treatment may diminish the concentration of human DNA and concentrate microorganisms. Nevertheless, the estimation of human DNA actin was not significantly different from other protocols. Although higher concentration of microbial DNA was observed in protocol 5, fewer bacterial and fungal taxa were identified, suggesting that the addition of a pre-treatment resulted in the loss of microorganisms. On the other hand, this may have resulted from the removal of microbial DNA from dead and/or broken-down microorganisms. No methodologies based on sequence analysis of amplicons from microbial markers (such as 16S rRNA and ITS genes) can differentiate living, dead, or ruptured bacteria, as all of these generate the same positive signals^28^. Here, the centrifugation performed during the pre-treatment step contributed to pellet the living microorganisms and remove other particles in the supernatant such as free DNA, resulting in the enrichment of living microbes. Although we did not show that the pre-treatment step decreases the proportion of dead microorganisms, the protocol 5 including the pre-treatment step could be a very important issue. In effect, controversy exists regarding the presence of living commensal bacteria in the lung since the detected DNA sequences may result of the breakdown of microorganisms and not living/reproducing microbial community members.

## Conclusion

Collectively, the most relevant findings of our study are that we to show that low microbial biomass samples such as lung tissue samples require particular care to avoid the risk of retrieving highly contaminated sequences. In addition, we found that the DNA extraction protocols affect the detection and abundance of bacterial and fungal taxa, which might have an influence on the interpretation of results, even though a small percentage of the total estimated microbial communities were affected. The bead-beating and Phenol:Chloroform:Isoamyl alcohol steps improve the detection of bacterial and fungal communities through an efficient lysis of microorganisms and a better quality of extracted DNA respectively. Additionally, we found that the addition of a pre-treatment step improves the amplification of microbial DNA, but also it might contribute to eliminate microbial DNA fragment resulting of dead microorganisms in lung tissue samples. Therefore, the microbial communities’ profiles determined in samples processed with the protocol 5 that includes the pre-treatment step might be closer to those inhabiting the lung. As lung microbiome studies are needed to determine whether pathogenic relationships between microbiome and lung disease exist, this protocol, including the pre-treatment step described, represent a reproducible alternative to avoid controversy on the live microorganisms inhabiting the lung.

## Methods

### Ethical issues

The Ethics Commission for Studies in Human Subjects of the University of Chile School of Medicine approved this study under protocol CEISH #092-2013. Informed consent was not obtained because this was a retrospective study of stored samples without identifiers as to the subjects of origin, which is in line with Chilean laws and regulations and assures protection of the subjects. All methods were performed in accordance with the principles of the Declaration of Helsinki for medical research involving human subjects and with the relevant local guidelines and regulations.

### Study design and sample collection

Chilean law requires autopsy for accidental deaths and for unexpected infant deaths. They are conducted at the Servicio Médico Legal de Santiago, which is the coroner’s office institution for the Metropolitan Area of Chile. Fresh frozen samples are kept stored after the autopsy procedure so they can be used for further analyses. Stored lung samples from legally required autopsies were selected based on unexpected death, storage at − 80 °C within less than 12 h from death, absence of previous admission to the hospital, and absence of known immunocompromising conditions and of obvious pulmonary disease at autopsy examination. Samples from four children aged 4.7 ± 2.8 months without an ascertainable cause of death and categorized as sudden infant deaths, and from three adults aged 42.4 ± 17.4 years whose death was categorized as road accident, were sent frozen to our laboratory without possible identifiers to link their identity.

For each subject, the right upper lobe was removed using sterile equipment and stored at − 80 °C in a sterile plastic bag until processing for analysis. The samples were removed from the bag and placed on a sterile plate in a biosafety cabinet. The pleura was removed to access untouched tissue using sterile equipment. Small samples were obtained from deep lung tissue, cut into small pieces (0.4 g), and frozen at – 80 °C.

### DNA extraction protocols

In this study, we compared five DNA extraction protocols (Table 1) based on modifications to the QIAamp DNA Mini kit (QIAGEN) used to detect *Pneumocystis*^29^. The protocols were as follows:

*Protocol 1.* Lung tissue was homogenized in 200-μl of PBS using the Ultra Turrax homogenizer (BIOSPEC PRODUCTS INC.). DNA was extracted and purified using the QIAamp DNA Mini kit (QIAGEN), according to the manufacturer's instructions.

*Protocol 2*. DNA extraction followed the procedure in protocol 1 with an additional bead-beating step to increase the disruption of microbial cells, using 400 μl of sterilized zirconia beads (0.1 and 0.5 mm) and homogenization using a Mini-Beadbeater-8 (2 × 3 min) (BIOSPEC PRODUCT INC.).

*Protocol 3.* This protocol supplemented the protocol 1 with an additional Phenol:Chloroform:Isoamyl alcohol (25:24:1) step after the lysis step performed using the QIAamp DNA Mini kit (QIAGEN). We recovered the upper aqueous phase to obtain the DNA.

*Protocol 4.* It consisted in the addition of both bead-beating and Phenol:Chloroform:Isoamyl alcohol steps to the protocol 1. After the last lysis step, we added 400 μl of sterilized zirconia beads and a volume of Phenol:Chloroform:Isoamyl alcohol (25:24:1). The upper aqueous solution was used to extract the DNA.

*Protocol 5.* This protocol was performed according to the procedure of the protocol 4. However, lung tissue is processed by a “Pre-treatment step”, consisting of homogenizing the lung by agitation with a magnetic stirrer in 20 ml of sterile PBS (pH 7.2) in ice pack–covered screw-capped flasks for 30 min, instead of with the Ultra Turrax homogenizer. The homogenate was filtered using sterile gauze and the stirring flasks were washed with sterile PBS to collect any remnants of the specimens. The filtrate was centrifuged and the pellet was reconstituted in 200 µl of sterile PBS, and DNA was extracted as in protocol 4.

### DNA extraction controls

Five blank samples consisting of buffer supplied with the kit were processed together with the lung tissue samples. Four were processed with the Ultra Turrax homogenizer and DNA was extracted according to the protocols 1–4, whereas the fifth was processed with the pre-treatment step according to the protocol 5.

### DNA quantitation and quality assessment

DNA concentrations were initially measured using the Qubit double-stranded DNA (dsDNA) BR assay kit on a Qubit fluorometer (INVITROGEN), and dilutions were accordingly prepared and measured. In addition, we determined DNA purity by measuring the concentration of undiluted DNA and absorbance ratios at 260/280 using a NanoDrop 1000 spectrophotometer (THERMO SCIENTIFIC).

### Estimation of human, bacterial and fungal DNA levels in DNA extracted from lung tissue samples

Levels of human, bacterial and fungal DNA were assessed using qPCR method by amplifying the human β-actin, the bacterial 16S rRNA, and the 18S rDNA gene of fungi, respectively. qPCRs were carried out in 10 μl reactions containing 2 μl of diluted template (~ 10 ng/μl) or water (negative template control), 2× LightCycler480 SYBR Green I Master (ROCHE DIAGNOSTICS), 0.3 μM each of the forward and reverse primers: 5′-TTGTTACAGGAAGTCCCTTGCC-3′ and 5′-ATGCTATCACCTCCCCTGTGTG-3′ to amplify the human β-actin; the forward 515F 5′-GTGCCAGCMGCCGCGGTAA-3′ and reverse 5′-CTTGTGCGGKCCCCCGYCAATTC-3′ to amplify the V4 hyper variable region of the 16S rRNA gene of bacteria^30^; and the forward 5′-TTAGCATGGAATAATRRAATAGGA-3′ and reverse 5′-TCTGGACCTGGTGAGTTTCC-3′ to amplify the V4 (partial) and V5 variable regions of the 18S rDNA of fungi^31^, and 2.4 μl of water. PCR reactions were performed on the Roche LightCycler 480 instrument (ROCHE DIAGNOSTIC). All samples were adjusted to 10 ng/µl using the Qubit Fluorometer (INVITROGEN), allowing for comparison of *C*t values.

### 16S rRNA gene and ITS amplification, library construction, and sequencing

For bacterial 16S rRNA gene amplification, primers (forward primer 5′-TCGTCGGCAGCGTCAGATGTGTATAAGAGACAGCCTACGGGNGGCWGCAG-3′ and reverse primer 5′-GTCTCGTGGGCTCGGAGATGTGTATAAGAGACAGGACTACHVGGGTATCTAATCC-3′) spanning the V3/V4 hypervariable regions were used. Internal controls of extraction and amplification were analysed together with the samples. As for fungi, an internal transcribed spacer (ITS) region was amplified. A pre-amplification with primers ITS1-F 5′-TAGAGGAAGTAAAAGTCGTAA-3′ and ITS2-R_KYO2 5′-TTYRCTRCGTTCTTCATC-3′ spanning the small subunit and the 5.8S region of the rRNA operon, was carried out. A second amplification with internal primers (ITS1-FInt 5′-*TCGTCGGCAGCGTCAGATGTGTATAAGAGACAG*GGAAGTAAAAGTCGTAACAAGG-3′, and ITS2_RInt: 5′-*GTCTCGTGGGCTCGGAGATGTGTATAAGAGACAG*CTRYGTTCTTCATCGDT-3′) containing the adapters sequence (in italics), was performed on 10.5 μl of the primary PCR. The resulting products were verified in a 1.4% agarose gel and purified amplicons were quantified using a Qubit Fluorometer (THERMO FISHER SCIENTIFIC). Next, dual indices were attached to both ends of the PCR products using Nextera XT Index Kit (ILLUMINA). Equimolar amounts of DNA per sample were pooled and on a MiSeq desktop sequencer (2 × 300 bp paired-end reads) (ILLUMINA).

### Bioinformatic processing

Forward and reverse fastq files containing reads in matched order, free of primer, adapter and linker sequences served as input for the DADA2 pipeline^32^, which analyses the quality profiles, filters and trims to remove Ns, expected errors and low quality tails. After learning the error rates with the DADA2 algorithm (https://benjjneb.github.io/dada2/tutorial.html), a dereplication step was used to reduce computation time. Paired reads were merged by aligning denoised forward and reverse reads, and merged reads served to construct the amplicon sequence variant table, to identify and remove chimeric sequences. Before taxonomy assignment, there was a removal of human sequences using bowtie2-2.3.4.2^33^ against the reference human genome database GRCh38.p11, using very sensitive parameters. The unaligned reads were used to assign taxonomy, using the Silva reference database for bacteria and Unite database for fungi. Finally, counts were obtained for operational taxonomic units (OTUs), and collapsed to the family level.

### Contaminated sequences assessment

The bacterial and fungal sequences clustering with the DADA2 pipeline at 100% identity with those present in the negative controls were removed from the analysis, for each group, respectively. The proportion of removed sequences was calculated for each sample.

### Bacterial and fungal community composition, abundance, and diversity analysis

The OTU table was converted into Biom format, using the QIIME pipeline version 1.9.0^34^ for composition and absolute and relative abundance analyses, as well as for ecological diversity. For alpha diversity, 1000 rarefactions with replacement were carried out and the Shannon diversity index was calculated. As for beta diversity, variation was assessed using canonical correspondence analysis (CCA), implemented in R version 3.1.0^35^, on a Bray–Curtis dissimilarity matrix.

### Statistics

Pairwise comparisons according to the method and individual were carried out using the qiime script compare_alpha_diveristy.py, with the default non-parametric t-tests. Boxplots were also generated by this script^36^. Fold change in relative abundance of family level taxa between distinct protocols was determined using the R DESEQ2 statistical software^37^ within the phyloseq package^38^. Wilcoxon-Mann–Whitney non-parametrical tests for groups of samples were conducted using R version 3.5.1^38^.

## Supplementary information

Supplementary Information.

## Data Availability

The sequence data are deposited in EBI Short Read Archive repository (https://www.ebi.ac.uk/ena) under study accession number PRJEB31011 with accession numbers for bacteria from ERS3088332 to ERS3088370 and for fungi from ERS3088371 to ERS3088409.
