# Supplementary material for: Optimized DNA extraction and purification method for characterization of bacterial and fungal communities in lung tissue samples
Source: Sci Rep. 2020 Oct 15;10:17377. doi: 10.1038/s41598-020-74137-2 (PMC7562954; doi:10.1038/s41598-020-74137-2)
Supplement: Supplementary file 1 — Supplementary Information. [file 41598_2020_74137_MOESM1_ESM.pdf]

Optimized DNA extraction and purification method for characterization of bacterial and fungal communities in lung tissue samples

Vicente Pérez-Brocal[1,2*], Fabien Magne[3,*#], Susana Ruiz-Ruiz[1,2*], Carolina A. Ponce[3], Rebeca Bustamante[3], Viviana San Martin[4], Mireya Gutierrez[4], Gianna Gatti[4], Sergio L. Vargas[3#] and Andrés Moya[1,2,5#]

[1]Department of Genomics and Health, Foundation for the Promotion of Health and Biomedical Research of Valencia Region (FISABIO-Public Health), València, Spain

[2]CIBER in Epidemiology and Public Health (CIBEResp), Madrid, Spain

[3]Microbiology and Mycology Program, Biomedical Sciences Institute (ICBM), University of Chile School of Medicine, Santiago, Chile

[4]Médico Legal Institute of Chile, Santiago, Chile

[5]Institute for Integrative Systems Biology (I2SysBio), University of Valencia and Spanish National Research Council (CSIC), València, Spain.

*These co-authors contributed equally to the work.

# Authors for correspondence:

Andrés Moya Email: Andres.Moya@uv.es

Fabien Magne Email: fabienmagne@med.uchile.cl

Sergio Vargas Email: svargas@med.uchile.cl

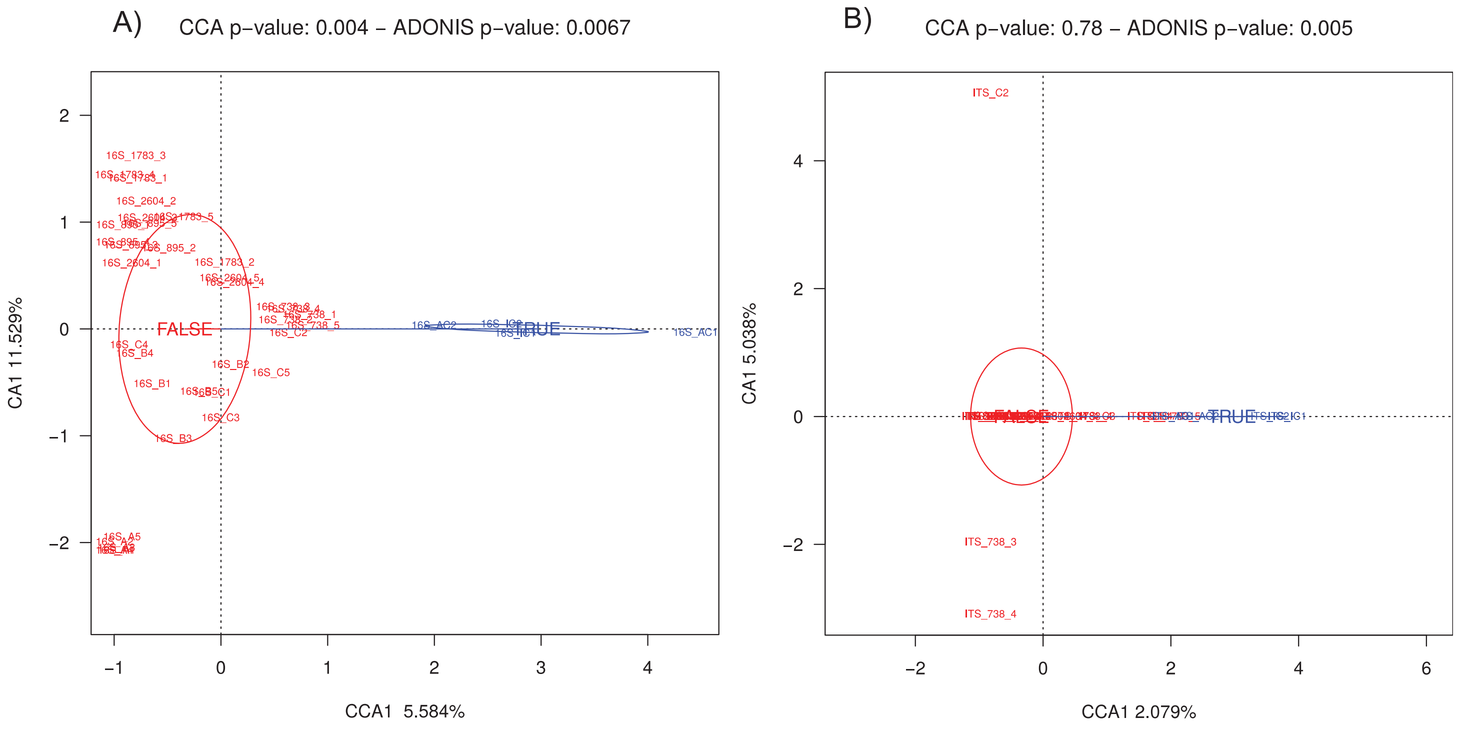

Supplemental Figure S1: CCA plots of bacterial (A) and fungal (B) microbiomes according to sample type. Red characters represent lung tissue samples. Blue characters are blank controls.

Supplemental Figure S2: Relative abundance of bacteria (A) and fungi (B) at phylum and familiy level for each lung tissue sample, according to the five extraction protocols described in this work. Sequencing of the 16S rRNA gene for bacteria and the ITS region for fungi carried out on 7 lung tissue samples using the Illumina MiSeq platform. Each protocol used for extraction is indicated as a digit (1 to 5) at the end of the sample name.

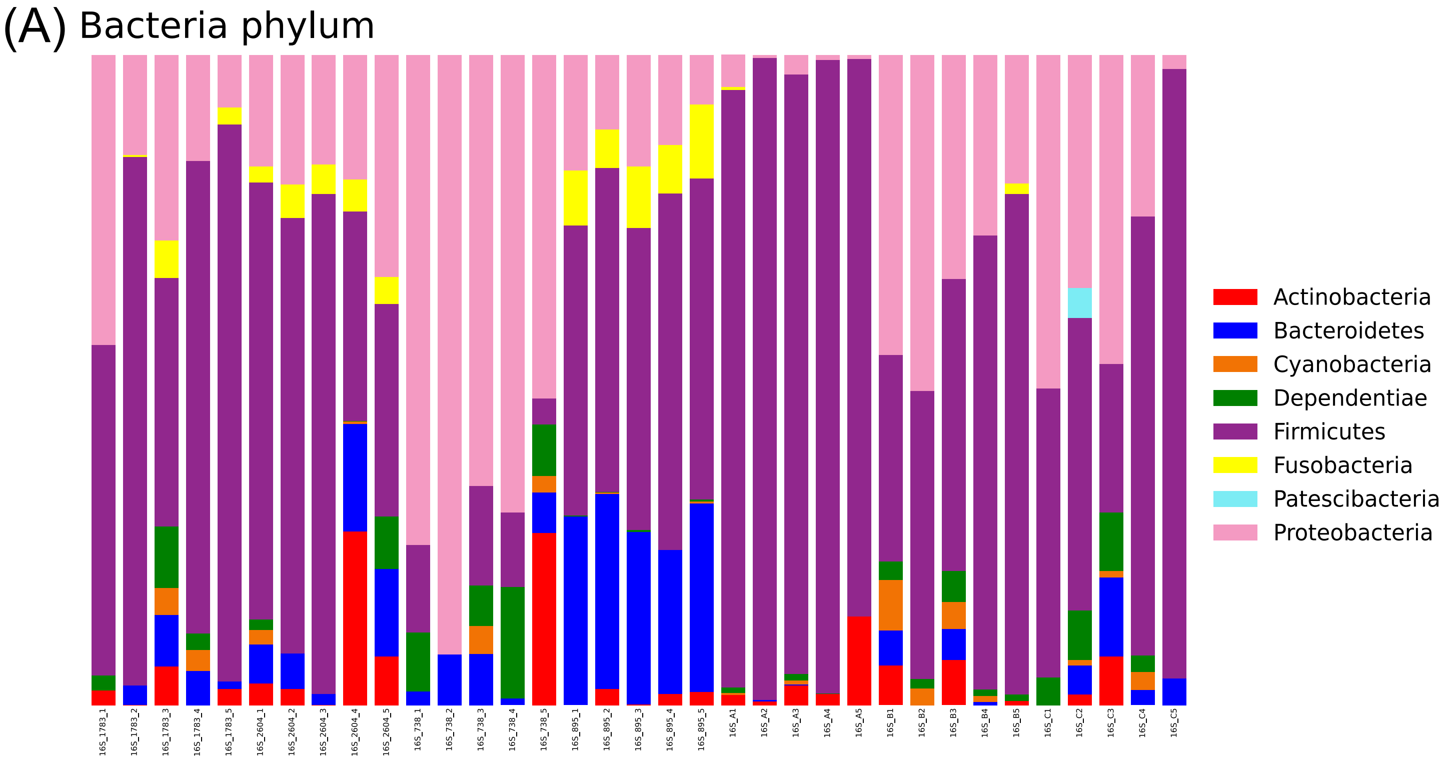

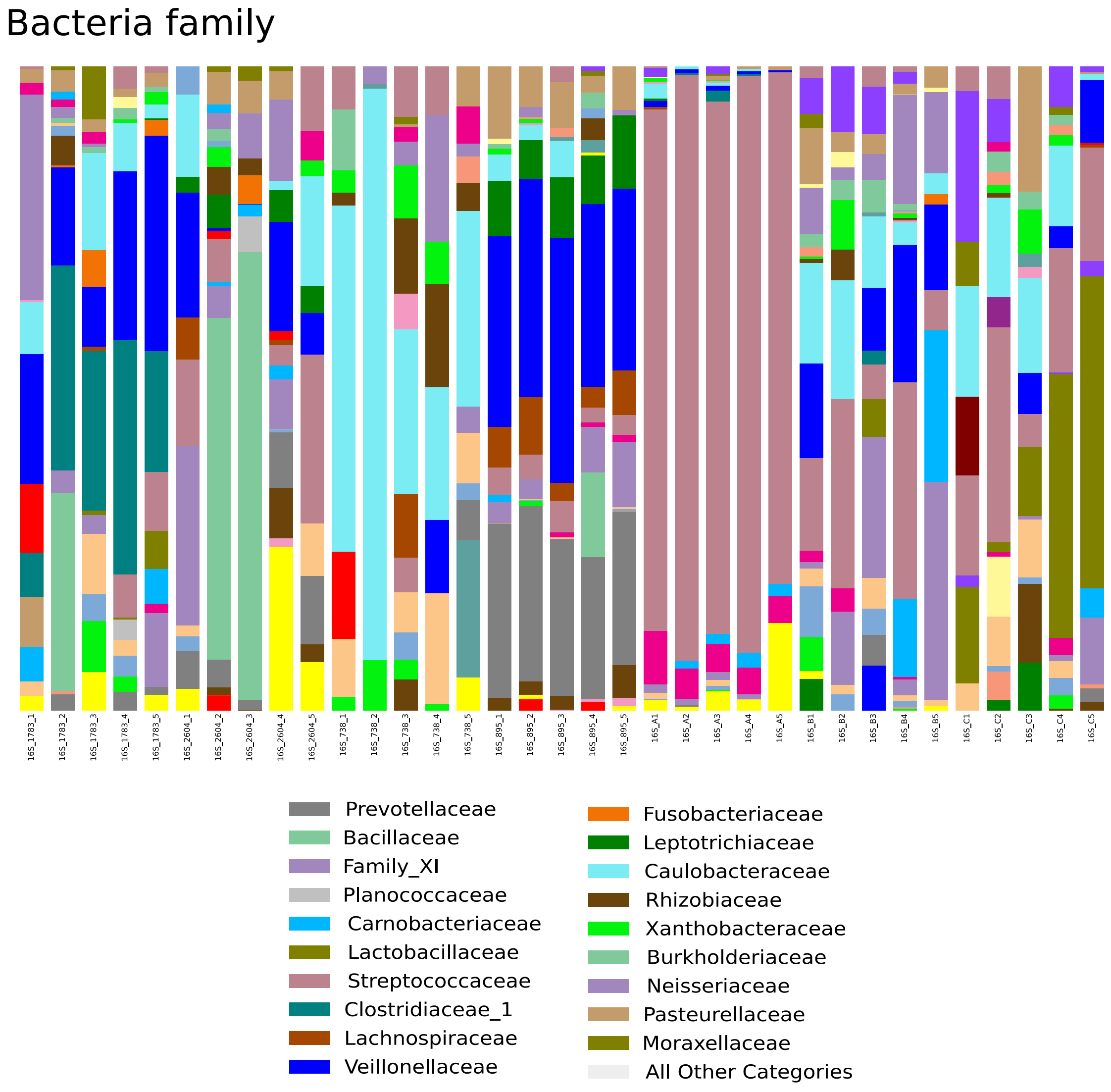

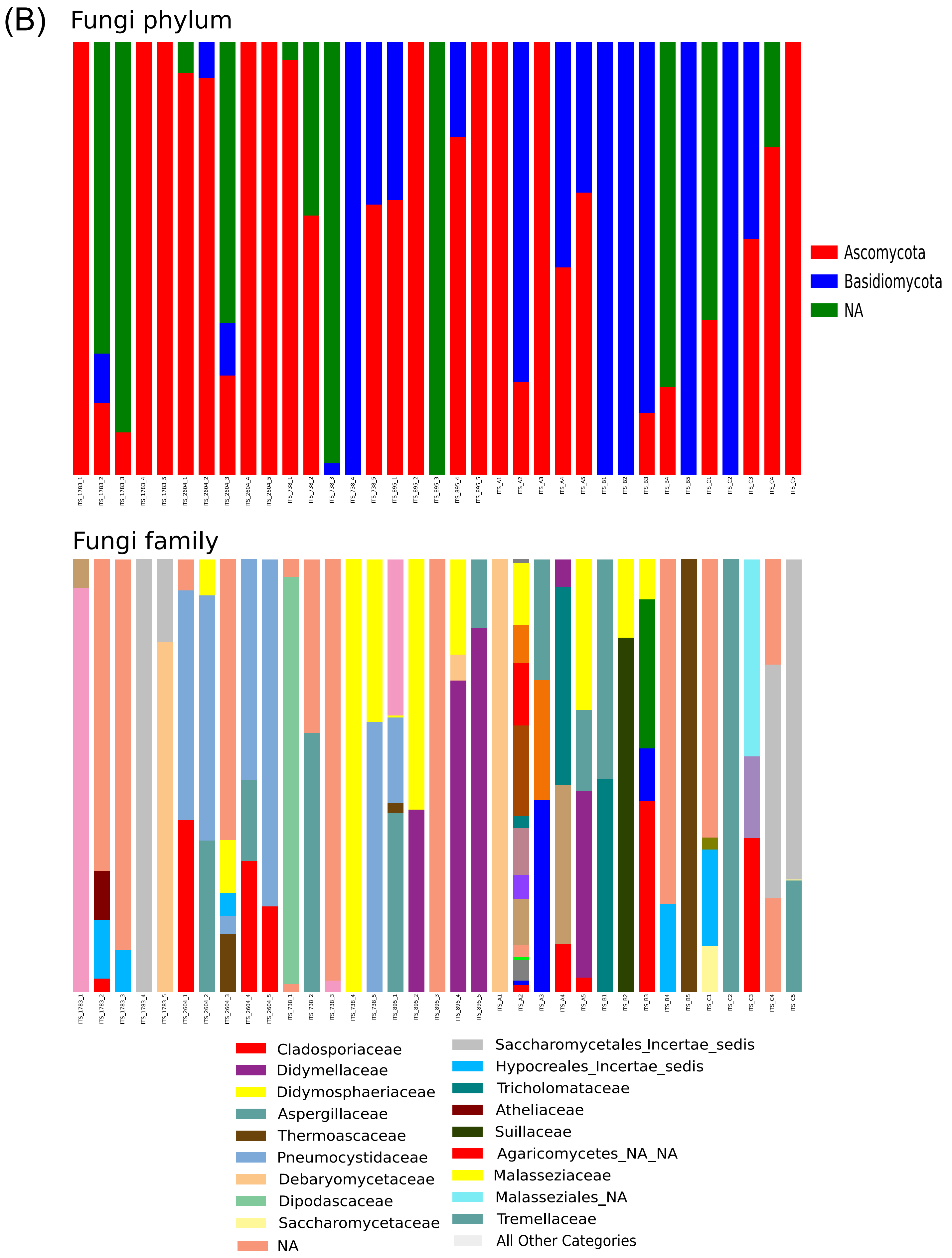
(B)
Fungi phylum
Ascomycota
Basidiomycota
NA
ITS_1783_1 ITS_1783_2 ITS_1783_3 ITS_1783_4 ITS_1783_5 ITS_2604_1 ITS_2604_2 ITS_2604_3 ITS_2604_4 ITS_2604_5 ITS_738_1 ITS_738_2 ITS_738_3 ITS_738_4 ITS_738_5 ITS_895_1 ITS_895_2 ITS_895_3 ITS_895_4 ITS_895_5 ITS_A1 ITS_A2 ITS_A3 ITS_A4 ITS_A5 ITS_B1 ITS_B2 ITS_B3 ITS_B4 ITS_B5 ITS_C1 ITS_C2 ITS_C3 ITS_C4 ITS_C5
Fungi family
ITS_1783_1 ITS_1783_2 ITS_1783_3 ITS_1783_4 ITS_1783_5 ITS_2604_1 ITS_2604_2 ITS_2604_3 ITS_2604_4 ITS_2604_5 ITS_738_1 ITS_738_2 ITS_738_3 ITS_738_4 ITS_738_5 ITS_895_1 ITS_895_2 ITS_895_3 ITS_895_4 ITS_895_5 ITS_A1 ITS_A2 ITS_A3 ITS_A4 ITS_A5 ITS_B1 ITS_B2 ITS_B3 ITS_B4 ITS_B5 ITS_C1 ITS_C2 ITS_C3 ITS_C4 ITS_C5
Cladosporiaceae
Didymellaceae
Didymosphaeriaceae
Aspergillaceae
Thermoascaceae
Pneumocystidaceae
Debaryomycetaceae
Dipodascaceae
Saccharomycetaceae
NA
Saccharomycetales_Incertae_sedis
Hypocreales_Incertae_sedis
Tricholomataceae
Atheliaceae
Suillaceae
Agaricomycetes_NA_NA
Malasseziaceae
Malasseziales_NA
Tremellaceae
All Other Categories

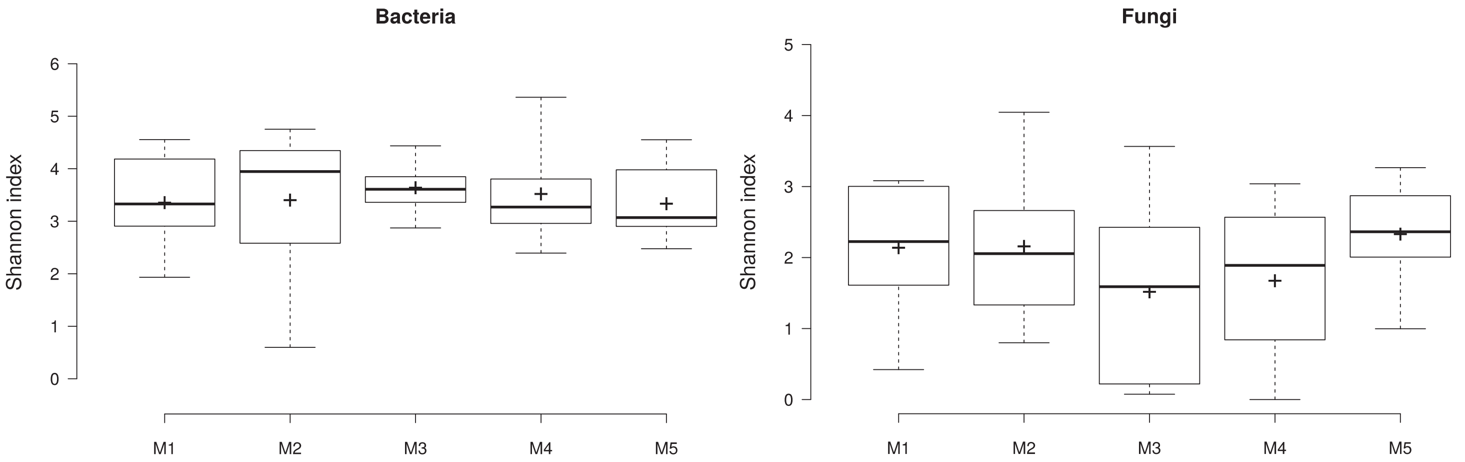

Group1	Group2	G1 mean	G1 std	G2 mean	G2 std	t stat	p-value
M1	M2	3.356	0.694	3.404	1.289	-0.08	0.931
M1	M3	3.356	0.694	3.64	0.348	-0.896	0.402
M1	M4	3.356	0.694	3.522	0.753	-0.396	0.729
M1	M5	3.356	0.694	3.336	0.561	0.055	0.959
M2	M3	3.404	1.289	3.64	0.348	-0.434	0.745
M2	M4	3.404	1.289	3.522	0.753	-0.194	0.888
M2	M5	3.404	1.289	3.336	0.561	0.118	0.911
M3	M4	3.64	0.348	3.522	0.753	0.35	0.741
M3	M5	3.64	0.348	3.336	0.561	1.129	0.283
M4	M5	3.522	0.753	3.336	0.561	0.485	0.642

Group1	Group2	G1 mean	G1 std	G2 mean	G2 std	t stat	p-value
M1	M2	2.14	0.821	2.158	0.936	-0.037	0.964
M1	M3	2.14	0.821	1.519	1.199	1.047	0.336
M1	M4	2.14	0.821	1.675	1.011	0.875	0.392
M1	M5	2.14	0.821	2.331	0.669	-0.443	0.644
M2	M3	2.158	0.936	1.519	1.199	1.03	0.333
M2	M4	2.158	0.936	1.675	1.011	0.86	0.407
M2	M5	2.158	0.936	2.331	0.669	-0.369	0.716
M3	M4	1.519	1.199	1.675	1.199	-0.244	0.8
M3	M5	1.519	1.199	2.331	0.669	-1.45	0.179
M4	M5	1.675	1.011	2.331	0.669	-1.327	0.242

Supplemental Figure S3: Boxplots showing the Shannon diversity index for each extraction protocol. M1 to M5: Protocols 1 to 5.

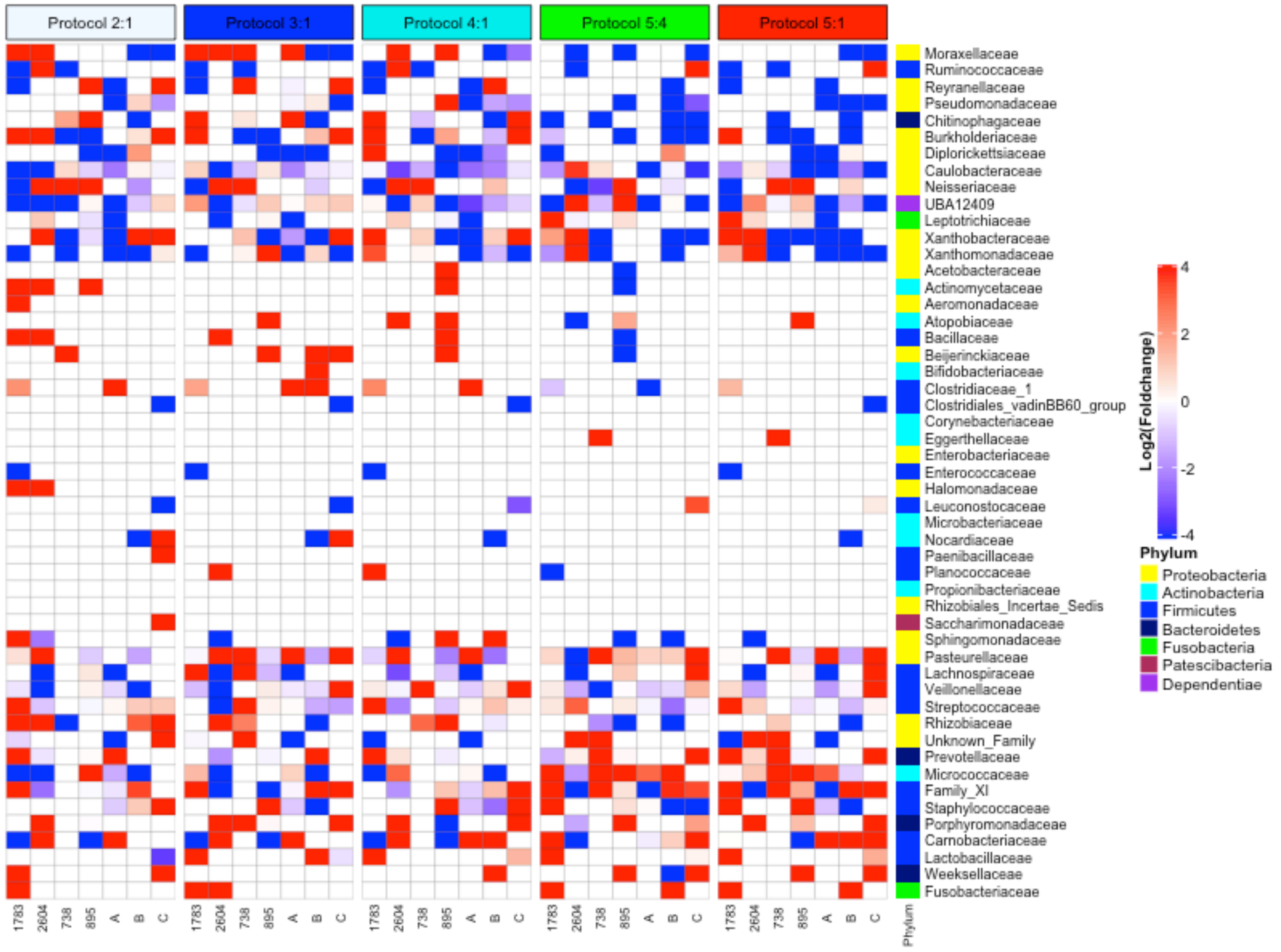

Supplemental Figure S4: Individual bacterial changes associated with bead beating (comparison of protocol 2 with 1); the Phenol:Chloroform:Isoamyl alcohol step (comparison of protocol 3 with 1); bead-beating and the Phenol:Chloroform:Isoamyl alcohol steps (comparison of protocol 4 with 1); pre-treatment steps (comparison of protocol 5 with 4); and pre-treatment, the bead-beating and the Phenol:Chloroform:Isoamyl alcohol steps (comparison of protocol 5 with 1). Log2 fold-change in relative taxon abundance was calculated using gtools package in R (https://www.rdocumentation.org/packages/gtools).

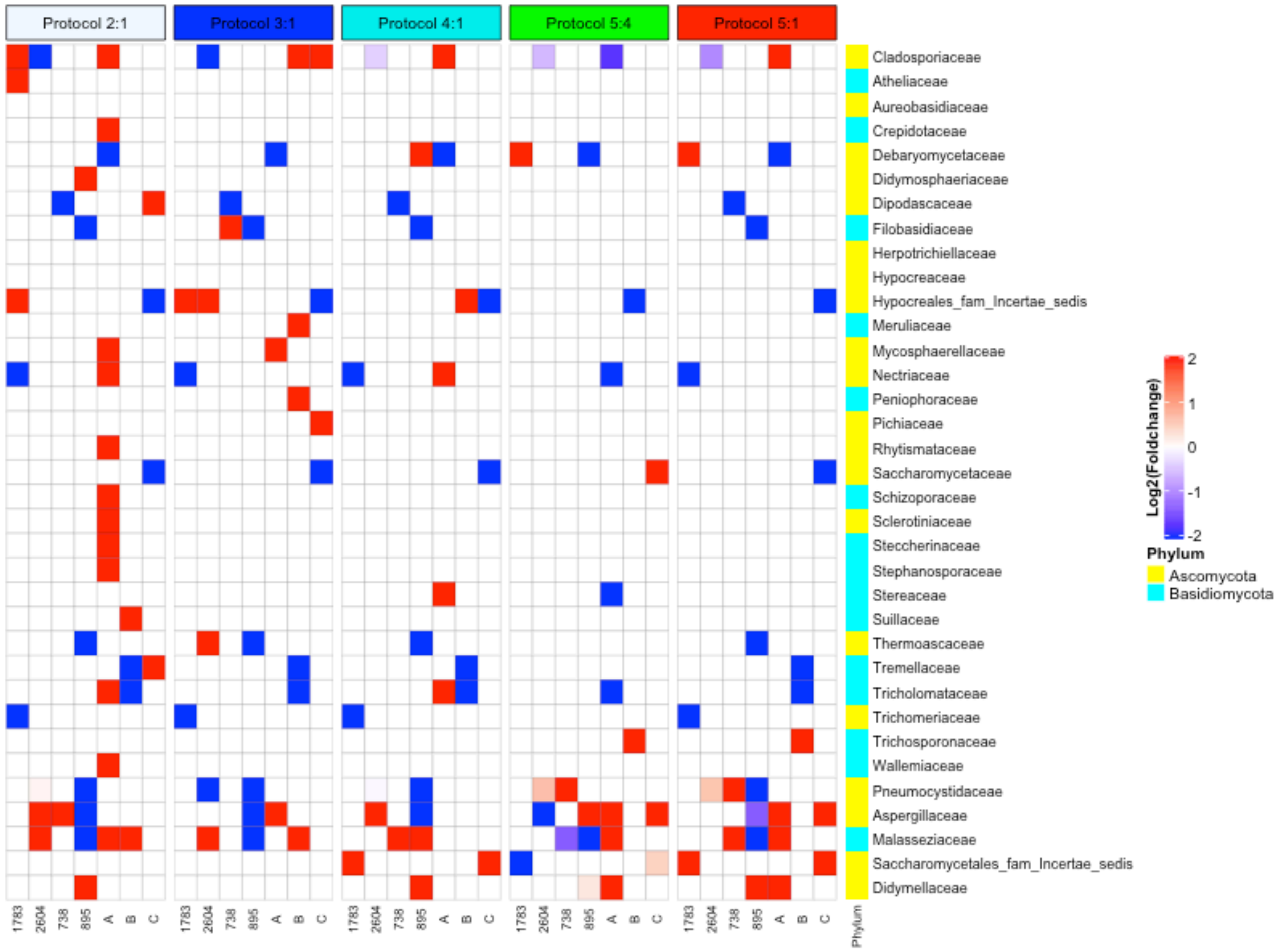

Supplemental Figure S5: Individual fungal changes associated with bead beating (comparison of protocol 2 with 1); the Phenol:Chloroform:Isoamyl alcohol step (comparison of protocol 3 with 1); bead-beating and the Phenol:Chloroform:Isoamyl alcohol steps (comparison of protocol 4 with 1); pre-treatment steps (comparison of protocol 5 with 4); and pre-treatment, the bead-beating and the Phenol:Chloroform:Isoamyl alcohol steps (comparison of protocol 5 with 1). Log2 fold-change in relative taxon abundance was calculated using gtools package in R (https://www.rdocumentation.org/packages/gtools).

Supplemental Table S1: Total count and percentage of bacterial and fungal families identified in negative controls during the DNA extraction process.

Bacteria

Family	counts	%
p__Euryarchaeota;c__Methanobacteria;o__Methanobacteriales;f__Methanobacteriaceae	44	0,19
p__Actinobacteria;c__Actinobacteria;o__Bifidobacteriales;f__Bifidobacteriaceae	20	0,09
p__Actinobacteria;c__Actinobacteria;o__Corynebacteriales;f__Corynebacteriaceae	153	0,67
p__Actinobacteria;c__Actinobacteria;o__Corynebacteriales;f__Dietziaceae	3	0,01
p__Actinobacteria;c__Actinobacteria;o__Corynebacteriales;f__Nocardiaceae	966	4,25
p__Actinobacteria;c__Actinobacteria;o__Frankiales;f__Geodermatophilaceae	18	0,08
p__Actinobacteria;c__Actinobacteria;o__Micrococcales;f__Microbacteriaceae	146	0,64
p__Actinobacteria;c__Actinobacteria;o__Micrococcales;f__Micrococcaceae	300	1,32
p__Actinobacteria;c__Actinobacteria;o__Propionibacteriales;f__Propionibacteriaceae	122	0,54
p__Actinobacteria;c__Coriobacteriia;o__Coriobacteriales;f__Coriobacteriaceae	2	0,01
p__Bacteroidetes;c__Bacteroidia;o__Bacteroidales;f__Bacteroidaceae	14	0,06
p__Bacteroidetes;c__Bacteroidia;o__Bacteroidales;f__Dysgonomonadaceae	14	0,06
p__Bacteroidetes;c__Bacteroidia;o__Bacteroidales;f__Paludibacteraceae	9	0,04
p__Bacteroidetes;c__Bacteroidia;o__Bacteroidales;f__Porphyromonadaceae	14	0,06
p__Bacteroidetes;c__Bacteroidia;o__Bacteroidales;f__Prevotellaceae	207	0,91
p__Bacteroidetes;c__Bacteroidia;o__Bacteroidales;f__Tannerellaceae	48	0,21
p__Bacteroidetes;c__Bacteroidia;o__Chitinophagales;f__Chitinophagaceae	120	0,53
p__Bacteroidetes;c__Bacteroidia;o__Cytophagales;f__Hymenobacteraceae	36	0,16
p__Bacteroidetes;c__Bacteroidia;o__Flavobacteriales;f__Weeksellaceae	206	0,91
p__Cyanobacteria;c__Melainabacteria;o__Obscuribacterales;f__NA	6	0,03
p__Deinococcus-Thermus;c__Deinococci;o__Deinococcales;f__Deinococcaceae	4	0,02
p__Firmicutes;c__Bacilli;o__Bacillales;f__Family_XI	158	0,69
p__Firmicutes;c__Bacilli;o__Bacillales;f__Staphylococcaceae	27	0,12
p__Firmicutes;c__Bacilli;o__Lactobacillales;f__Carnobacteriaceae	53	0,23
p__Firmicutes;c__Bacilli;o__Lactobacillales;f__Enterococcaceae	149	0,65
p__Firmicutes;c__Bacilli;o__Lactobacillales;f__Streptococcaceae	1.949	8,56
p__Firmicutes;c__Clostridia;o__Clostridiales;f__Clostridiaceae_1	21	0,09
p__Firmicutes;c__Clostridia;o__Clostridiales;f__Family_XI	118	0,52
p__Firmicutes;c__Clostridia;o__Clostridiales;f__Lachnospiraceae	34	0,15
p__Firmicutes;c__Clostridia;o__Clostridiales;f__Peptostreptococcaceae	34	0,15
p__Firmicutes;c__Clostridia;o__Clostridiales;f__Ruminococcaceae	146	0,64
p__Firmicutes;c__Erysipelotrichia;o__Erysipelotrichales;f__Erysipelotrichaceae	45	0,20
p__Firmicutes;c__Negativicutes;o__Selenomonadales;f__Veillonellaceae	194	0,85
p__Fusobacteria;c__Fusobacteriia;o__Fusobacteriales;f__Fusobacteriaceae	113	0,50
p__Fusobacteria;c__Fusobacteriia;o__Fusobacteriales;f__Leptotrichiaceae	50	0,22
p__NA;c__NA;o__NA;f__NA	29	0,13
p__Proteobacteria;c__Alphaproteobacteria;o__Caulobacterales;f__Caulobacteraceae	93	0,41
p__Proteobacteria;c__Alphaproteobacteria;o__Rhizobiales;f__Beijerinckiaceae	7	0,03
p__Proteobacteria;c__Alphaproteobacteria;o__Rhizobiales;f__Devosiaceae	20	0,09
p__Proteobacteria;c__Alphaproteobacteria;o__Rhizobiales;f__Hyphomicrobiaceae	54	0,24
p__Proteobacteria;c__Alphaproteobacteria;o__Rhizobiales;f__Rhizobiaceae	5.549	24,38
p__Proteobacteria;c__Alphaproteobacteria;o__Rhizobiales;f__Rhizobiales_Incertae_Sedis	174	0,76
p__Proteobacteria;c__Alphaproteobacteria;o__Rhizobiales;f__Xanthobacteraceae	83	0,36
p__Proteobacteria;c__Alphaproteobacteria;o__Rhodospirillales;f__Rhodospirillaceae	30	0,13
p__Proteobacteria;c__Alphaproteobacteria;o__Sphingomonadales;f__Sphingomonadaceae	226	0,99
p__Proteobacteria;c__Gammaproteobacteria;o__Betaproteobacteriales;f__Burkholderiaceae	611	2,69
p__Proteobacteria;c__Gammaproteobacteria;o__Betaproteobacteriales;f__Neisseriaceae	585	2,57
p__Proteobacteria;c__Gammaproteobacteria;o__Enterobacteriales;f__Enterobacteriaceae	167	0,73
p__Proteobacteria;c__Gammaproteobacteria;o__Gammaproteobacteria_Incertae_Sedis;f__Unknown_Family	15	0,07
p__Proteobacteria;c__Gammaproteobacteria;o__Pasteurellales;f__Pasteurellaceae	29	0,13
p__Proteobacteria;c__Gammaproteobacteria;o__Pseudomonadales;f__Moraxellaceae	498	2,19
p__Proteobacteria;c__Gammaproteobacteria;o__Pseudomonadales;f__Pseudomonadaceae	8.749	38,45
p__Proteobacteria;c__Gammaproteobacteria;o__Xanthomonadales;f__Rhodanobacteraceae	6	0,03
p__Proteobacteria;c__Gammaproteobacteria;o__Xanthomonadales;f__Xanthomonadaceae	246	1,08
p__WPS-2;c__NA;o__NA;f__NA	42	0,18
Total	22.756	100

Fungi

Family	counts	%
p__Ascomy	4.523	6,19
p__Ascomy	3.270	4,48
p__Ascomy	443	0,61
p__Ascomy	2.367	3,24
p__Ascomy	39.349	53,88
p__Ascomy	78	0,11
p__Ascomy	109	0,15
p__Ascomy	304	0,42
p__Ascomy	12.517	17,14
p__Basidior	6.562	8,98
p__Basidior	166	0,23
p__Chytridi	166	0,23
p__NA;c__N	3.183	4,36
Total	73.037	100

Supplemental Table S2: Total counts and percentages of bacterial and fungal families identified in lung tissue samples.

Bacteria

Family	counts	%
p__Actinobacteria;c__Actinobacteria;o__Actinomycetales;f__Actinomycetaceae	1.363	0,65
p__Actinobacteria;c__Actinobacteria;o__Bifidobacteriales;f__Bifidobacteriaceae	63	0,03
p__Actinobacteria;c__Actinobacteria;o__Corynebacteriales;f__Corynebacteriaceae	0	0,00
p__Actinobacteria;c__Actinobacteria;o__Corynebacteriales;f__Nocardiaceae	240	0,11
p__Actinobacteria;c__Actinobacteria;o__Micrococcales;f__Microbacteriaceae	0	0,00
p__Actinobacteria;c__Actinobacteria;o__Micrococcales;f__Micrococcaceae	1.525	0,73
p__Actinobacteria;c__Actinobacteria;o__Propionibacteriales;f__Propionibacteriaceae	0	0,00
p__Actinobacteria;c__Coriobacteriia;o__Coriobacteriales;f__Atopobiaceae	218	0,10
p__Actinobacteria;c__Coriobacteriia;o__Coriobacteriales;f__Eggerthellaceae	149	0,07
p__Bacteroidetes;c__Bacteroidia;o__Bacteroidales;f__Porphyromonadaceae	1.254	0,60
p__Bacteroidetes;c__Bacteroidia;o__Bacteroidales;f__Prevotellaceae	14.112	6,73
p__Bacteroidetes;c__Bacteroidia;o__Chitinophagales;f__Chitinophagaceae	413	0,20
p__Bacteroidetes;c__Bacteroidia;o__Flavobacteriales;f__Weeksellaceae	388	0,19
p__Cyanobacteria;c__Melainabacteria;o__Obscuribacterales;f__NA	601	0,29
p__Dependentiae;c__Babeliae;o__Babeliales;f__UBA12409	1.181	0,56
p__Firmicutes;c__Bacilli;o__Bacillales;f__Bacillaceae	60.086	28,68
p__Firmicutes;c__Bacilli;o__Bacillales;f__Family_XI	8.391	4,00
p__Firmicutes;c__Bacilli;o__Bacillales;f__Paenibacillaceae	225	0,11
p__Firmicutes;c__Bacilli;o__Bacillales;f__Planococcaceae	1.829	0,87
p__Firmicutes;c__Bacilli;o__Bacillales;f__Staphylococcaceae	1.260	0,60
p__Firmicutes;c__Bacilli;o__Lactobacillales;f__Carnobacteriaceae	2.326	1,11
p__Firmicutes;c__Bacilli;o__Lactobacillales;f__Enterococcaceae	113	0,05
p__Firmicutes;c__Bacilli;o__Lactobacillales;f__Lactobacillaceae	3.393	1,62
p__Firmicutes;c__Bacilli;o__Lactobacillales;f__Leuconostocaceae	129	0,06
p__Firmicutes;c__Bacilli;o__Lactobacillales;f__Streptococcaceae	22.115	10,55
p__Firmicutes;c__Clostridia;o__Clostridiales;f__Clostridiaceae_1	16.267	7,76
p__Firmicutes;c__Clostridia;o__Clostridiales;f__Clostridiales_vadinBB60_group	140	0,07
p__Firmicutes;c__Clostridia;o__Clostridiales;f__Family_XI	0	0,00
p__Firmicutes;c__Clostridia;o__Clostridiales;f__Lachnospiraceae	2.095	1,00
p__Firmicutes;c__Clostridia;o__Clostridiales;f__Ruminococcaceae	760	0,36
p__Firmicutes;c__Negativicutes;o__Selenomonadales;f__Veillonellaceae	24.770	11,82
p__Fusobacteria;c__Fusobacteriia;o__Fusobacteriales;f__Fusobacteriaceae	1.768	0,84
p__Fusobacteria;c__Fusobacteriia;o__Fusobacteriales;f__Leptotrichiaceae	5.537	2,64
p__Patescibacteria;c__Saccharimonadia;o__Saccharimonadales;f__Saccharimonadaceae	112	0,05
p__Proteobacteria;c__Alphaproteobacteria;o__Acetobacterales;f__Acetobacteraceae	166	0,08
p__Proteobacteria;c__Alphaproteobacteria;o__Caulobacterales;f__Caulobacteraceae	4.312	2,06
p__Proteobacteria;c__Alphaproteobacteria;o__Reyranellales;f__Reyranellaceae	138	0,07
p__Proteobacteria;c__Alphaproteobacteria;o__Rhizobiales;f__Beijerinckiaceae	637	0,30
p__Proteobacteria;c__Alphaproteobacteria;o__Rhizobiales;f__Rhizobiaceae	6.020	2,87
p__Proteobacteria;c__Alphaproteobacteria;o__Rhizobiales;f__Rhizobiales_Incertae_Sedis	0	0,00
p__Proteobacteria;c__Alphaproteobacteria;o__Rhizobiales;f__Xanthobacteraceae	1.670	0,80
p__Proteobacteria;c__Alphaproteobacteria;o__Rhodospirillales;f__NA	195	0,09
p__Proteobacteria;c__Alphaproteobacteria;o__Sphingomonadales;f__Sphingomonadaceae	1.550	0,74
p__Proteobacteria;c__Gammaproteobacteria;o__Aeromonadales;f__Aeromonadaceae	215	0,10
p__Proteobacteria;c__Gammaproteobacteria;o__Betaproteobacteriales;f__Burkholderiaceae	2.346	1,12
p__Proteobacteria;c__Gammaproteobacteria;o__Betaproteobacteriales;f__Neisseriaceae	6.130	2,93
p__Proteobacteria;c__Gammaproteobacteria;o__Diplorickettsiales;f__Diplorickettsiaceae	109	0,05
p__Proteobacteria;c__Gammaproteobacteria;o__Enterobacteriales;f__Enterobacteriaceae	0	0,00
p__Proteobacteria;c__Gammaproteobacteria;o__Gammaproteobacteria_Incertae_Sedis;f__Unknown_Family	734	0,35
p__Proteobacteria;c__Gammaproteobacteria;o__Oceanospirillales;f__Halomonadaceae	1.041	0,50
p__Proteobacteria;c__Gammaproteobacteria;o__Pasteurellales;f__Pasteurellaceae	7.726	3,69
p__Proteobacteria;c__Gammaproteobacteria;o__Pseudomonadales;f__Moraxellaceae	1.812	0,86
p__Proteobacteria;c__Gammaproteobacteria;o__Pseudomonadales;f__Pseudomonadaceae	1.280	0,61
p__Proteobacteria;c__Gammaproteobacteria;o__Xanthomonadales;f__Xanthomonadaceae	629	0,30
Total	209.533	100

Fungi

Family	counts	%
p__Ascomy	45.438	2,56
p__Ascomy	3.395	0,19
p__Ascomy	1.963	0,11
p__Ascomy	63.873	3,59
p__Ascomy	22.573	1,27
p__Ascomy	5.898	0,33
p__Ascomy	66.311	3,73
p__Ascomy	24.760	1,39
p__Ascomy	1.220	0,07
p__Ascomy	158	0,01
p__Ascomy	2.714	0,15
p__Ascomy	65.623	3,69
p__Ascomy	42.260	2,38
p__Ascomy	33.431	1,88
p__Ascomy	5.843	0,33
p__Ascomy	15.314	0,86
p__Ascomy	51.193	2,88
p__Ascomy	116.573	6,56
p__Ascomy	6.988	0,39
p__Ascomy	3.870	0,22
p__Basidion	1.402	0,08
p__Basidion	2.753	0,15
p__Basidion	14.489	0,82
p__Basidion	17.353	0,98
p__Basidion	19.688	1,11
p__Basidion	5.310	0,30
p__Basidion	12.110	0,68
p__Basidion	3.445	0,19
p__Basidion	2.234	0,13
p__Basidion	9.790	0,55
p__Basidion	668	0,04
p__Basidion	62.585	3,52
p__Basidion	14.087	0,79
p__Basidion	9.066	0,51
p__Basidion	28.307	1,59
p__Basidion	4.477	0,25
p__Basidion	222	0,01
p__NA;c__N	989.587	55,69
Total	1.776.971	100

Supplemental Table S3. Percentage of bacterial and fungal families identified per sample in lung tissue samples, grouped by individual and by extraction method. Green color intensity is proportional to the relative abundance of a family in each sample.

Bacteria	Grouped per subject																																							
Family	1783					2604					738					895					A					B					C				
	1783_1	1783_2	1783_3	1783_4	1783_5	2604_1	2604_2	2604_3	2604_4	2604_5	738_1	738_2	738_3	738_4	738_5	895_1	895_2	895_3	895_4	895_5	A1	A2	A3	A4	A5	B1	B2	B3	B4	B5	C1	C2	C3	C4	C5
Actinomycetaceae	0	0,07	0	0	0	0	2,4	0	0	0	0	0	0	0	0	0	1,77	0	1,36	0	0	0	0	0	0	0	0	0	0	0	0	0	0	0	0
Bifidobacteriaceae	0	0	0	0	0	0	0	0	0	0	0	0	0	0	0	0	0	0	0	0	0	0	0	0	0	0	0	6,99	0	0	0	0	0	0	0
Corynebacteriaceae	0	0	0	0	0	0	0	0	0	0	0	0	0	0	0	0	0	0	0	0	0	0	0	0	0	0	0	0	0	0	0	0	0	0	0
Nocardiaceae	0	0	0	0	0	0	0	0	0	0	0	0	0	0	0	0	0	0	0	0	0	0	0	0	0	4,92	0	0	0	0	0	1,65	7,49	0	0
Microbacteriaceae	0	0	0	0	0	0	0	0	0	0	0	0	0	0	0	0	0	0	0	0	0	0	0	0	0	0	0	0	0	0	0	0	0	0	0
Micrococcaceae	2,31	0	6	0	2,5	3,37	0,09	0,02	25,51	7,54	0	0	0	0	5,16	0	0,74	0	0	0,69	1,59	0,6	3,01	1,75	13,67	1,23	0	0	0	0,71	0	0	0	0	0
Propionibacteriaceae	0	0	0	0	0	0	0	0	0	0	0	0	0	0	0	0	0	0	0	0	0	0	0	0	0	0	0	0	0	0	0	0	0	0	0
Atopobiaceae	0	0	0	0	0	0	0	0	1,26	0	0	0	0	0	0	0	0	0,13	0,42	1,36	0	0	0	0	0	0	0	0	0	0	0	0	0	0	0
Eggerthellaceae	0	0	0	0	0	0	0	0	0	0	0	0	0	0	21,38	0	0	0	0	0	0	0	0	0	0	0	0	0	0	0	0	0	0	0	0
Porphyromonadaceae	0	0	0	0	0	0	1,1	0,05	7,81	2,79	0	0	4,83	0	0	2,03	2,02	2,21	0	5,02	0	0	0	0	0	0	0	0	0	0	0	0	12,21	0,35	1,32
Prevotellaceae	0	2,46	0	2,95	1,2	5,99	4,34	1,65	8,67	10,61	0	0	0	0	6,17	27,02	27,25	24,34	22,11	23,8	0	0,25	0	0	0	0	0	4,77	0	0	0	0	0	0	2,17
Chitinophagaceae	0	0	7,93	2,36	0	0	0	0	0	0	2,19	7,85	3,07	1,07	0	0	0,74	0	0	0	0	0	0,21	0	0	5,37	0	0	0,32	0	0	0	0	2,03	0
Weeksellaceae	0	0,5	0	0	0	0	0	0	0	0	0	0	0	0	0	0	0	0	0	0,13	0	0	0	0	0	0	0	0	0,22	0	0	4,47	0	0	0,6
NA	0	0	4,16	3,2	0	2,24	0	0	0,43	0	0	0	4,29	0	2,58	0	0,18	0	0	0,33	0,29	0	0,63	0	0	7,79	2,59	4,11	0,9	0	0	0,87	0,98	2,73	0
UBA12409	2,25	0	9,39	2,53	0	1,62	0	0	0,09	8,1	9	0	6,21	17,14	7,89	0,13	0,14	0,27	0	0,31	0,9	0	0,95	0,09	0	2,78	1,47	4,77	0,96	1,01	4,27	7,61	9,03	2,55	0
Bacillaceae	0	30,8	0	0	0	0	53,04	69,54	0	0	0	0	0	0	0	0	0	0	13,12	0	0	0	0	0	0	0	0	0	0	0	0	0	0	0	0
Family_XI	0	3,48	2,93	0	11,45	27,93	5,05	0	7,68	0	0	0	0	0	4,02	3,19	3,05	0	7,05	10,09	1,3	0,98	1,19	0,75	0	1	11,31	21,86	2,48	33,84	0	0	0,57	0,99	10,38
Paenibacillaceae	0	0	0	0	0	0	0	0	0	0	0	0	0	0	0	0	0	0	0	0	0	0	0	0	0	0	0	0	0	0	0	9,31	0	0	0
Planococcaceae	0	0	0	3,12	0	0	0	5,5	0	0	0	0	0	0	0	0	0	0	0	0	0	0	0	0	0	0	0	0	0	0	0	0	0	0	0
Staphylococcaceae	0	0	0	0	1,46	0	0	0	0	0	0	0	0	0	0	0	0	0,71	0,72	1,11	8,32	4,69	4,38	4,09	4,16	1,73	3,63	0	0,32	0	0	0,74	0	2,67	0
Carnobacteriaceae	5,38	0	0	0	5,41	0	0,5	1,86	2,17	0	0	0	0	0	0	1,08	0	0	0	0	0	1,2	1,61	2,26	1,86	0	0	0	12,14	23,51	0	0	0	0	4,52
Enterococcaceae	7,69	0	0	0	0	0	0	0	0	0	0	0	0	0	0	0	0	0	0	0	0	0	0	0	0	0	0	0	0	0	0	0	0	0	0
Lactobacillaceae	0	0	0,69	0,34	5,88	0	0	0	0	0	0	0	0	0	0	0	0	0	0	0	0	0	0	0	0	0	0	5,88	0	0	15	1,49	10,66	41,01	48,49
Leuconostocaceae	0	0	0	0	0	0	0	0	0	0	0	0	0	0	0	0	0	0	0	0	0	0	0	0	0	0	0	0	0	0	1,74	0	0	0,23	2,35
Streptococcaceae	0	0,02	0	6,66	9,21	13,34	6,66	0	3,12	26,26	0	0	5,37	0	0	4,3	3,9	4,91	2,28	3,06	80,93	90,9	82,59	89,68	79,43	14,44	29,36	5,33	33,65	6,18	15,52	33,43	5,13	19,26	17,61
Clostridiaceae_1	6,94	31,79	24,79	36,39	18,69	0	0	0	0	0	0	0	0	0	0	0	0	0	0	0	0	0,35	1,75	0,16	0	0	0	2,22	0	0	0	0	0	0	0
Clostridiales_vadinBB60_group	0	0	0	0	0	0	0	0	0	0	0	0	0	0	0	0	0	0	0	0	0	0	0	0	0	0	0	0	0	0	12,21	0	0	0	0
Family_XI	0	0	0	0	0	0	0	0	0	0	0	0	0	0	0	0	0	0	0	0	0	0	0	0	0	0	0	0	0	0	0	0	0	0	0
Lachnospiraceae	0	0	0,69	0	0	6,61	0	0	0,78	0	0	0	9,97	0	0	6,33	8,89	2,79	3,21	6,9	0,43	0	0	0	0	0	0	0	0	0	0	0	0	0	0,4
Ruminococcaceae	10,62	0	0	0	0	0	1,29	0	1,34	0	13,5	0	0	0	0	0	0	0	0	0	0	0	0	0	0	0	0	0	0	0	0	0	0	0	0,31
Veillonellaceae	20,22	15,21	9,16	26,2	33,52	19,33	0,5	0,04	17,05	6,42	0	0	0	11,43	0	29,68	33,91	38,07	28,45	28,21	0,9	0,57	0,7	0,51	0,28	14,66	0	9,66	21,26	13,37	0	0	6,43	3,42	9,73
Fusobacteriaceae	0	0,33	5,77	0	2,39	0	0	4,53	0	0	0	0	0	0	0	0	0	0	0	0	0	0	0	0	0	0	0	0	0	1,62	0	0	0	0	0
Leptotrichiaceae	0	0	0	0	0,26	2,49	5,14	0,02	4,95	4,19	0	0	0	0	0	8,52	5,99	9,43	7,49	11,42	0,4	0	0	0	0	0	0	0	0	0	0	0	0	0	0
Saccharimonadaceae	0	0	0	0	0	0	0	0	0	0	0	0	0	0	0	0	0	0	0	0	0	0	0	0	0	0	0	0	0	0	0	4,63	0	0	0
Acetobacteraceae	0	0	0	0	0	0	0	0	0	0	0	0	0	0	0	0	0	0	0,52	0	0	0	0	0	0	0	0	0	0	0	0	0	0	0	0
Caulobacteraceae	8,03	0	15,09	7,58	2,13	12,72	0	0	1,39	17,04	53,73	88,74	25,46	20,54	30,42	4,07	2,34	5,58	0	0	2,24	0,44	0,53	0,37	0	15,66	18,48	11,21	3,6	3,24	17,18	15,43	14,73	12,47	0,92
Reyranellaceae	0,34	0	0	0	0	0	0	0	0	0	0	0	5,52	0	0	0	0,28	0	0	0	0,33	0	0,28	0	0	0	0	0	0,26	0	0	0,08	1,71	0	0
Beijerinckiaceae	0	0	0	0	0	0	0	0	0	0	0	0,68	0	0	0	0	0	0,58	1,85	0	0	0	0	0	0	0	0	0,55	0	0	0	0	2,03	0	0
Rhizobiaceae	0	4,6	0	0	0	0	4,35	2,55	0	0	2,06	0	11,73	16,07	4,3	0	0	0	3,43	0	0	0	0	0	0	0,55	4,75	0	0,42	0	0	0,62	0	0	0
Rhizobiales_Incertae_Sedis	0	0	0	0	0	0	0	0	0	0	0	0	0	0	0	0	0	0	0	0	0	0	0	0	0	0	0	0	0	0	0	0	0	0	0
Xanthobacteraceae	0	0	0	0,51	1,93	0	3,09	0	0	2,51	3,47	0	8,21	6,61	0	0,98	0,67	0	0	0	0,58	0	0,18	0	0	0,36	7,68	0	0,7	0	0	1,28	6,84	1,68	0
NA	0	0	0	0	0	0	0	0	0	0	0	0	0	0	4,16	0	0,28	1,46	0	0	0	0	0	0	0	1,5	0	0	0,3	0	0	2,07	0	1,57	0
Sphingomonadaceae	0	1,53	0	0	0	4,36	0,86	0	0	0	0	0	0	0	0	0	0	0	1,5	0	0	0	0	0	0	0	0	0	0,22	0	0	0	0	0	0
Aeromonadaceae	0	0,46	0	0	0	0	0	0	0	0	0	0	0	0	0	0	0	0	0	0	0	0	0	0	0	0	0	0	0	0	0	0	0	0	0
Burkholderiaceae	0	0,83	0,92	1,77	0,83	0	1,98	0	0	0	9,38	0	0	0	0	0,69	0	0	2,5	0	0	0	0	0	0	2,09	3,11	5,11	0,92	0	0	3,1	2,77	1,57	0
Neisseriaceae	31,86	1,65	0,54	0	0	0	2,44	7,01	12,67	0	0	2,73	3,68	19,64	2,01	0	1,59	0	0	0,82	0	0	0	0	0	7,1	1,99	4	16,88	12,56	0	0	0	0	0
Diplorickettsiaceae	0	0	0	1,68	0	0	0	0	0	0	0	0	0	0	0	0,77	0	0	0	0	0,14	0	0	0	0	0,59	2,42	0	0,14	0,71	0	0	0	0	0
Enterobacteriaceae	0	0	0	0	0	0	0	0	0	0	0	0	0	0	0	0	0	0	0	0	0	0	0	0	0	0	0	0	0	0	0	0	0	0	0
Unknown_Family	1,91	1,19	1,77	0	0	0	0	0	0	4,47	0	0	2,22	0	5,74	0	0	0	0	0	0,11	0	0	0	0	0	0	0	0	0	0	1,45	0	0	0
Halomonadaceae	0	1,2	0	0	0	0	1,35	0	0	0	0	0	0	0	0	0	0	0	0	0	0	0	0	0	0	0	0	0	0	0	0	0	0	0	0
Pasteurellaceae	2,11	3,28	2	1,26	2,19	0	5,01	5,08	4,34	0	0	0	0,46	0	6,17	11,2	6,24	7,08	2,48	6,72	0	0	0,6	0,33	0,59	8,74	3,02	3,11	1,64	3,24	0	0	19,45	0	0,36
Moraxellaceae	0	0,61	8,16	0	0	0	0,81	2,15	0,74	0	0	0	1,15	0	0	0	0	0	0,76	0	0	0	0,21	0	0	2,19	0	0	0	0	6,89	0	0	1,22	0
Pseudomonadaceae	0	0	0	0	0	0	0	0	0	0	0	0	0	0	0	0	0	0	0,74	0	1,34	0	1,19	0	0	5,46	10,19	7,33	1,86	0	23,37	6,74	0	6,26	0,85
Xanthomonadaceae	0,34	0	0	3,45	0,94	0	0	0	0	10,06	6,68	0	7,82	7,5	0	0	0	2,43	0	0	0,18	0	0	0	0	1,82	0	3,11	0,8	0	3,84	5,01	0	0	0

Fungi — Grouped per subject

Family	1783_1	1783_2	1783_3	1783_4	1783_5	2604_1	2604_2	2604_3	2604_4	2604_5	738_1	738_2	738_3	738_4	738_5
	**1783**					**2604**					**738**				
Cladosporiaceae	0	3,11	0	0	0	39,72	0	0	30,27	19,8	0	0	0	0	0
Mycosphaerellaceae	0	0	0	0	0	0	0	0	0	0	0	0	0	0	0
o__Capnodiales;f__NA	0	0	0	0	0	0	0	0	0	0	0	0	0	0	0
Didymellaceae	0	0	0	0	0	0	0	0	0	0	0	0	0	0	0
Didymosphaeriaceae	0	0	0	0	0	0	0	0	0	0	0	0	0	0	0
Trichomeriaceae	93,44	0	0	0	0	0	0	0	0	0	0	0	0	0	0
Aspergillaceae	0	0	0	0	0	0	35,05	0	18,81	0	0	59,88	0	0	0
Thermoascaceae	0	0	0	0	0	0	0	13,45	0	0	0	0	0	0	0
Sclerotiniaceae	0	0	0	0	0	0	0	0	0	0	0	0	0	0	0
Rhytismataceae	0	0	0	0	0	0	0	0	0	0	0	0	0	0	0
p__Ascomycota;c__NA;o__NA;f__NA	0	0	0	0	0	0	0	0	0	0	1,79	0	0	0	0
Pneumocystidaceae	0	0	0	0	0	53,15	56,66	4,08	50,92	80,2	0	0	0	0	62,42
Debaryomycetaceae	0	0	0	0	80,93	0	0	0	0	0	0	0	0	0	0
Dipodascaceae	0	0	0	0	0	0	0	0	0	0	94,13	0	0	0	0
Pichiaceae	0	0	0	0	0	0	0	0	0	0	0	0	0	0	0
Saccharomycetaceae	0	0	0	0	0	0	0	0	0	0	0	0	0	0	0
Saccharomycetales_fam_Incertae_sedis	0	0	0	100	19,07	0	0	0	0	0	0	0	0	0	0
Hypocreales_fam_Incertae_sedis	0	13,51	9,73	0	0	0	0	5,36	0	0	0	0	0	0	0
Nectriaceae	6,56	0	0	0	0	0	0	0	0	0	0	0	0	0	0
c__Sordariomycetes;o__NA;f__NA	0	0	0	0	0	0	0	0	0	0	0	0	0	0	0
Crepidotaceae	0	0	0	0	0	0	0	0	0	0	0	0	0	0	0
Stephanosporaceae	0	0	0	0	0	0	0	0	0	0	0	0	0	0	0
Tricholomataceae	0	0	0	0	0	0	0	0	0	0	0	0	0	0	0
Atheliaceae	0	11,4	0	0	0	0	0	0	0	0	0	0	0	0	0
Suillaceae	0	0	0	0	0	0	0	0	0	0	0	0	0	0	0
Schizoporaceae	0	0	0	0	0	0	0	0	0	0	0	0	0	0	0
c__Agaricomycetes;o__NA;f__NA	0	0	0	0	0	0	0	0	0	0	0	0	0	0	0
Meruliaceae	0	0	0	0	0	0	0	0	0	0	0	0	0	0	0
Steccherinaceae	0	0	0	0	0	0	0	0	0	0	0	0	0	0	0
Peniophoraceae	0	0	0	0	0	0	0	0	0	0	0	0	0	0	0
Stereaceae	0	0	0	0	0	0	0	0	0	0	0	0	0	0	0
Malasseziaceae	0	0	0	0	0	0	8,3	12,2	0	0	0	0	0	100	37,58
o__Malasseziales;f__NA	0	0	0	0	0	0	0	0	0	0	0	0	0	0	0
Filobasidiaceae	0	0	0	0	0	0	0	0	0	0	0	0	2,65	0	0
Tremellaceae	0	0	0	0	0	0	0	0	0	0	0	0	0	0	0
Trichosporonaceae	0	0	0	0	0	0	0	0	0	0	0	0	0	0	0
Wallemiaceae	0	0	0	0	0	0	0	0	0	0	0	0	0	0	0
k__Fungi;p__NA;c__NA;o__NA;f__NA	0	71,98	90,27	0	0	7,13	0	64,92	0	0	4,08	40,12	97,35	0	0

Family	895_1	895_2	895_3	895_4	895_5	A1	A2	A3	A4	A5	B1	B2	B3	B4	B5	C1	C2	C3	C4	C5
	**895**					**A**					**B**					**C**				
Cladosporiaceae	0	0	0	0	0	0	1,59	0	11,11	3,33	0	0	14,27	0	0	0	0	35,6	0	0
Mycosphaerellaceae	0	0	0	0	0	0	1,02	44,4	0	0	0	0	0	0	0	0	0	0	0	0
o__Capnodiales;f__NA	0	0	0	0	0	0	0	27,78	0	0	0	0	0	0	0	0	0	0	0	0
Didymellaceae	0	42,17	0	71,99	84,25	0	0	0	0	43,1	0	0	0	0	0	0	0	0	0	0
Didymosphaeriaceae	0	57,84	0	0	0	0	0	0	0	0	0	0	0	0	0	0	0	0	0	0
Trichomeriaceae	0	0	0	0	0	0	0	0	0	0	0	0	0	0	0	0	0	0	0	0
Aspergillaceae	41,34	0	0	0	15,75	0	0	27,82	0	18,78	0	0	0	0	0	0	0	0	0	25,78
Thermoascaceae	2,34	0	0	0	0	0	0	0	0	0	0	0	0	0	0	0	0	0	0	0
Sclerotiniaceae	0	0	0	0	0	0	4,84	0	0	0	0	0	0	0	0	0	0	0	0	0
Rhytismataceae	0	0	0	0	0	0	0,63	0	0	0	0	0	0	0	0	0	0	0	0	0
p__Ascomycota;c__NA;o__NA;f__NA	0	0	0	0	0	0	2,84	0	0	0	0	0	0	0	0	0	0	0	21,79	0
Pneumocystidaceae	19,77	0	0	0	0	0	0	0	0	0	0	0	0	0	0	0	0	0	0	0
Debaryomycetaceae	0	0	0	6,05	0	100	0	0	0	0	0	0	0	0	0	0	0	0	0	0
Dipodascaceae	0	0	0	0	0	0	0	0	0	0	0	0	0	0	0	0	0,02	0	0	0
Pichiaceae	0	0	0	0	0	0	0	0	0	0	0	0	0	0	0	0	0	18,88	0	0
Saccharomycetaceae	0	0	0	0	0	0	0	0	0	0	0	0	0	0	0	10,59	0	0	0	0,28
Saccharomycetales_fam_Incertae_sedis	0	0	0	0	0	0	0	0	0	0	0	0	0	0	0	0	0	0	53,91	73,94
Hypocreales_fam_Incertae_sedis	0	0	0	0	0	0	0	0	0	0	0	0	0	20,3	0	22,4	0	0	0	0
Nectriaceae	0	0	0	0	0	0	10,55	0	36,77	0	0	0	0	0	0	0	0	0	0	0
c__Sordariomycetes;o__NA;f__NA	0	0	0	0	0	0	0	0	0	0	0	0	0	0	0	2,7	0	0	0	0
Crepidotaceae	0	0	0	0	0	0	5,56	0	0	0	0	0	0	0	0	0	0	0	0	0
Stephanosporaceae	0	0	0	0	0	0	10,91	0	0	0	0	0	0	0	0	0	0	0	0	0
Tricholomataceae	0	0	0	0	0	0	2,69	0	45,84	0	49,26	0	0	0	0	0	0	0	0	0
Atheliaceae	0	0	0	0	0	0	0	0	0	0	0	0	0	0	0	0	0	0	0	0
Suillaceae	0	0	0	0	0	0	0	0	0	0	0	81,91	0	0	0	0	0	0	0	0
Schizoporaceae	0	0	0	0	0	0	21,05	0	0	0	0	0	0	0	0	0	0	0	0	0
c__Agaricomycetes;o__NA;f__NA	0	0	0	0	0	0	14,34	0	0	0	0	0	29,9	0	0	0	0	0	0	0
Meruliaceae	0	0	0	0	0	0	0	0	0	0	0	0	12,13	0	0	0	0	0	0	0
Steccherinaceae	0	0	0	0	0	0	8,85	0	0	0	0	0	0	0	0	0	0	0	0	0
Peniophoraceae	0	0	0	0	0	0	0	0	0	0	0	0	34,47	0	0	0	0	0	0	0
Stereaceae	0	0	0	0	0	0	0	0	6,28	0	0	0	0	0	0	0	0	0	0	0
Malasseziaceae	0,5	0	0	21,96	0	0	14,26	0	0	34,79	0	18,09	9,23	0	0	0	0	0	0	0
o__Malasseziales;f__NA	0	0	0	0	0	0	0	0	0	0	0	0	0	0	0	0	0	45,52	0	0
Filobasidiaceae	36,06	0	0	0	0	0	0	0	0	0	0	0	0	0	0	0	0	0	0	0
Tremellaceae	0	0	0	0	0	0	0	0	0	0	50,74	0	0	0	0	0	99,98	0	0	0
Trichosporonaceae	0	0	0	0	0	0	0	0	0	0	0	0	0	0	100	0	0	0	0	0
Wallemiaceae	0	0	0	0	0	0	0,88	0	0	0	0	0	0	0	0	0	0	0	0	0
k__Fungi;p__NA;c__NA;o__NA;f__NA	0	0	100	0	0	0	0	0	0	0	0	0	0	79,7	0	64,31	0	0	24,3	0

Bacteria	Grouped per method																																		
Family	M1							M2							M3							M4							M5						
	1783_1	2604_1	738_1	895_1	A1	B1	C1	1783_2	2604_2	738_2	895_2	A2	B2	C2	1783_3	2604_3	738_3	895_3	A3	B3	C3	1783_4	2604_4	738_4	895_4	A4	B4	C4	1783_5	2604_5	738_5	895_5	A5	B5	C5
Actinomycetaceae	0	0	0	0	0	0	0	0,07	2,4	0	1,77	0	0	0	0	0	0	0	0	0	0	0	0	0	1,36	0	0	0	0	0	0	0	0	0	0
Bifidobacteriaceae	0	0	0	0	0	0	0	0	0	0	0	0	0	0	0	0	0	0	0	6,99	0	0	0	0	0	0	0	0	0	0	0	0	0	0	0
Corynebacteriaceae	0	0	0	0	0	0	0	0	0	0	0	0	0	0	0	0	0	0	0	0	0	0	0	0	0	0	0	0	0	0	0	0	0	0	0
Nocardiaceae	0	0	0	0	0	4,92	0	0	0	0	0	0	0	1,65	0	0	0	0	0	0	7,49	0	0	0	0	0	0	0	0	0	0	0	0	0	0
Microbacteriaceae	0	0	0	0	0	0	0	0	0	0	0	0	0	0	0	0	0	0	0	0	0	0	0	0	0	0	0	0	0	0	0	0	0	0	0
Micrococcaceae	2,31	3,37	0	0	1,59	1,23	0	0	0,09	0	0,74	0,6	0	0	6	0,02	0	0	3,01	0	0	0	25,51	0	0	1,75	0	0	2,5	7,54	5,16	0,69	13,67	0,71	0
Propionibacteriaceae	0	0	0	0	0	0	0	0	0	0	0	0	0	0	0	0	0	0	0	0	0	0	0	0	0	0	0	0	0	0	0	0	0	0	0
Atopobiaceae	0	0	0	0	0	0	0	0	0	0	0	0	0	0	0	0	0	0,13	0	0	0	0	1,26	0	0,42	0	0	0	0	0	0	1,36	0	0	0
Eggerthellaceae	0	0	0	0	0	0	0	0	0	0	0	0	0	0	0	0	0	0	0	0	0	0	0	0	0	0	0	0	0	0	21,38	0	0	0	0
Porphyromonadaceae	0	0	0	2,03	0	0	0	0	1,1	0	2,02	0	0	0	0	0,05	4,83	2,21	0	0	12,21	0	7,81	0	0	0	0	0,35	0	2,79	0	5,02	0	0	1,32
Prevotellaceae	0	5,99	0	27,02	0	0	0	2,46	4,34	0	27,25	0,25	0	0	0	1,65	0	24,34	0	4,77	0	2,95	8,67	0	22,11	0	0	0	1,2	10,61	6,17	23,8	0	0	2,17
Chitinophagaceae	0	0	2,19	0	0	5,37	0	0	0	7,85	0,74	0	0	0	7,93	0	3,07	0	0,21	0	0	2,36	0	1,07	0	0	0,32	2,03	0	0	0	0	0	0	0
Weeksellaceae	0	0	0	0	0	0	0	0,5	0	0	0	0	0	4,47	0	0	0	0	0	0	0	0	0	0	0	0	0,22	0	0	0	0	0,13	0	0	0,6
NA	0	2,24	0	0	0,29	7,79	0	0	0	0	0,18	0	2,59	0,87	4,16	0	4,29	0	0,63	4,11	0,98	3,2	0,43	0	0	0	0,9	2,73	0	0	2,58	0,33	0	0	0
UBA12409	2,25	1,62	9	0,13	0,9	2,78	4,27	0	0	0	0,14	0	1,47	7,61	9,39	0	6,21	0,27	0,95	4,77	9,03	2,53	0,09	17,14	0	0,09	0,96	2,55	0	8,1	7,89	0,31	0	1,01	0
Bacillaceae	0	0	0	0	0	0	0	30,8	53,04	0	0	0	0	0	0	69,54	0	0	0	0	0	0	0	0	13,12	0	0	0	0	0	0	0	0	0	0
Family_XI	0	27,93	0	3,19	1,3	1	0	3,48	5,05	0	3,05	0,98	11,31	0	2,93	0	0	0	1,19	21,86	0,57	0	7,68	0	7,05	0,75	2,48	0,99	11,45	0	4,02	10,09	0	33,84	10,38
Paenibacillaceae	0	0	0	0	0	0	0	0	0	0	0	0	0	9,31	0	0	0	0	0	0	0	0	0	0	0	0	0	0	0	0	0	0	0	0	0
Planococcaceae	0	0	0	0	0	0	0	0	0	0	0	0	0	0	0	5,5	0	0	0	0	0	3,12	0	0	0	0	0	0	0	0	0	0	0	0	0
Staphylococcaceae	0	0	0	0	8,32	1,73	0	0	0	0	0	4,69	3,63	0,74	0	0	0	0,71	4,38	0	0	0	0	0	0,72	4,09	0,32	2,67	1,46	0	0	1,11	4,16	0	0
Carnobacteriaceae	5,38	0	0	1,08	0	0	0	0	0,5	0	0	1,2	0	0	0	1,86	0	0	1,61	0	0	0	2,17	0	0	2,26	12,14	0	5,41	0	0	0	1,86	23,51	4,52
Enterococcaceae	7,69	0	0	0	0	0	0	0	0	0	0	0	0	0	0	0	0	0	0	0	0	0	0	0	0	0	0	0	0	0	0	0	0	0	0
Lactobacillaceae	0	0	0	0	0	0	15	0	0	0	0	0	0	1,49	0,69	0	0	0	0	5,88	10,66	0,34	0	0	0	0	0	41,01	5,88	0	0	0	0	0	48,49
Leuconostocaceae	0	0	0	0	0	0	1,74	0	0	0	0	0	0	0	0	0	0	0	0	0	0	0	0	0	0	0	0	0,23	0	0	0	0	0	0	2,35
Streptococcaceae	0	13,34	0	4,3	80,93	14,44	15,52	0,02	6,66	0	3,9	90,9	29,36	33,43	0	0	5,37	4,91	82,59	5,33	5,13	6,66	3,12	0	2,28	89,68	33,65	19,26	9,21	26,26	0	3,06	79,43	6,18	17,61
Clostridiaceae_1	6,94	0	0	0	0	0	0	31,79	0	0	0	0,35	0	0	24,79	0	0	0	1,75	2,22	0	36,39	0	0	0	0,16	0	0	18,69	0	0	0	0	0	0
Clostridiales_vadinBB60_group	0	0	0	0	0	0	12,21	0	0	0	0	0	0	0	0	0	0	0	0	0	0	0	0	0	0	0	0	0	0	0	0	0	0	0	0
Family_XI	0	0	0	0	0	0	0	0	0	0	0	0	0	0	0	0	0	0	0	0	0	0	0	0	0	0	0	0	0	0	0	0	0	0	0
Lachnospiraceae	0	6,61	0	6,33	0,43	0	0	0	0	0	8,89	0	0	0	0,69	0	9,97	2,79	0	0	0	0	0,78	0	3,21	0	0	0	0	0	0	6,9	0	0	0,4
Ruminococcaceae	10,62	0	13,5	0	0	0	0	0	1,29	0	0	0	0	0	0	0	0	0	0	0	0	0	1,34	0	0	0	0	0	0	0	0	0	0	0	0,31
Veillonellaceae	20,22	19,33	0	29,68	0,9	14,66	0	15,21	0,5	0	33,91	0,57	0	0	9,16	0,04	0	38,07	0,7	9,66	6,43	26,2	17,05	11,43	28,45	0,51	21,26	3,42	33,52	6,42	0	28,21	0,28	13,37	9,73
Fusobacteriaceae	0	0	0	0	0	0	0	0,33	0	0	0	0	0	0	5,77	4,53	0	0	0	0	0	0	0	0	0	0	0	0	2,39	0	0	0	0	1,62	0
Leptotrichiaceae	0	2,49	0	8,52	0,4	0	0	0	5,14	0	5,99	0	0	0	0	0,02	0	9,43	0	0	0	0	4,95	0	7,49	0	0	0	0,26	4,19	0	11,42	0	0	0
Saccharimonadaceae	0	0	0	0	0	0	0	0	0	0	0	0	0	4,63	0	0	0	0	0	0	0	0	0	0	0	0	0	0	0	0	0	0	0	0	0
Acetobacteraceae	0	0	0	0	0	0	0	0	0	0	0	0	0	0	0	0	0	0	0	0	0	0	0	0	0,52	0	0	0	0	0	0	0	0	0	0
Caulobacteraceae	8,03	12,72	53,73	4,07	2,24	15,66	17,18	0	0	88,74	2,34	0,44	18,48	15,43	15,09	0	25,46	5,58	0,53	11,21	14,73	7,58	1,39	20,54	0	0,37	3,6	12,47	2,13	17,04	30,42	0	0	3,24	0,92
Reyranellaceae	0,34	0	0	0	0,33	0	0	0	0	0	0,28	0	0	0,08	0	0	5,52	0	0,28	0	1,71	0	0	0	0	0	0,26	0	0	0	0	0	0	0	0
Beijerinckiaceae	0	0	0	0	0	0	0	0	0	0,68	0	0	0	0	0	0	0	0,58	0	0,55	2,03	0	0	0	1,85	0	0	0	0	0	0	0	0	0	0
Rhizobiaceae	0	0	2,06	0	0	0,55	0	4,6	4,35	0	0	0	4,75	0,62	0	2,55	11,73	0	0	0	0	0	0	16,07	3,43	0	0,42	0	0	0	4,3	0	0	0	0
Rhizobiales_Incertae_Sedis	0	0	0	0	0	0	0	0	0	0	0	0	0	0	0	0	0	0	0	0	0	0	0	0	0	0	0	0	0	0	0	0	0	0	0
Xanthobacteraceae	0	0	3,47	0,98	0,58	0,36	0	0	3,09	0	0,67	0	7,68	1,28	0	0	8,21	0	0,18	0	6,84	0,51	0	6,61	0	0	0,7	1,68	1,93	2,51	0	0	0	0	0
NA	0	0	0	0	0	1,5	0	0	0	0	0,28	0	0	2,07	0	0	0	1,46	0	0	0	0	0	0	0	0	0,3	1,57	0	0	4,16	0	0	0	0
Sphingomonadaceae	0	4,36	0	0	0	0	0	1,53	0,86	0	0	0	0	0	0	0	0	0	0	0	0	0	0	0	1,5	0	0,22	0	0	0	0	0	0	0	0
Aeromonadaceae	0	0	0	0	0	0	0	0,46	0	0	0	0	0	0	0	0	0	0	0	0	0	0	0	0	0	0	0	0	0	0	0	0	0	0	0
Burkholderiaceae	0	0	9,38	0,69	0	2,09	0	0,83	1,98	0	0	0	3,11	3,1	0,92	0	0	0	0	5,11	2,77	1,77	0	0	2,5	0	0,92	1,57	0,83	0	0	0	0	0	0
Neisseriaceae	31,86	0	0	0	0	7,1	0	1,65	2,44	2,73	1,59	0	1,99	0	0,54	7,01	3,68	0	0	4	0	0	12,67	19,64	0	0	16,88	0	0	0	2,01	0,82	0	12,56	0
Diplorickettsiaceae	0	0	0	0,77	0,14	0,59	0	0	0	0	0	0	2,42	0	0	0	0	0	0	0	0	1,68	0	0	0	0	0,14	0	0	0	0	0	0	0,71	0
Enterobacteriaceae	0	0	0	0	0	0	0	0	0	0	0	0	0	0	0	0	0	0	0	0	0	0	0	0	0	0	0	0	0	0	0	0	0	0	0
Unknown_Family	1,91	0	0	0	0,11	0	0	1,19	0	0	0	0	0	1,45	1,77	0	2,22	0	0	0	0	0	0	0	0	0	0	0	0	4,47	5,74	0	0	0	0
Halomonadaceae	0	0	0	0	0	0	0	1,2	1,35	0	0	0	0	0	0	0	0	0	0	0	0	0	0	0	0	0	0	0	0	0	0	0	0	0	0
Pasteurellaceae	2,11	0	0	11,2	0	8,74	0	3,28	5,01	0	6,24	0	3,02	0	2	5,08	0,46	7,08	0,6	3,11	19,45	1,26	4,34	0	2,48	0,33	1,64	0	2,19	0	6,17	6,72	0,59	3,24	0,36
Moraxellaceae	0	0	0	0	0	2,19	6,89	0,61	0,81	0	0	0	0	0	8,16	2,15	1,15	0	0,21	0	0	0	0,74	0	0,76	0	0	1,22	0	0	0	0	0	0	0
Pseudomonadaceae	0	0	0	0	1,34	5,46	23,37	0	0	0	0	0	10,19	6,74	0	0	0	0	1,19	7,33	0	0	0	0	0,74	0	1,86	6,26	0	0	0	0	0	0	0,85
Xanthomonadaceae	0,34	0	6,68	0	0,18	1,82	3,84	0	0	0	0	0	0	5,01	0	0	7,82	2,43	0	3,11	0	3,45	0	7,5	0	0	0,8	0	0,94	10,06	0	0	0	0	0

Fungi	Grouped per method																																		
Family	M1							M2							M3							M4							M5						
	1783_1	2604_1	738_1	895_1	A1	B1	C1	1783_2	2604_2	738_2	895_2	A2	B2	C2	1783_3	2604_3	738_3	895_3	A3	B3	C3	1783_4	2604_4	738_4	895_4	A4	B4	C4	1783_5	2604_5	738_5	895_5	A5	B5	C5
Cladosporiaceae	0	39,72	0	0	0	0	0	3,11	0	0	0	1,59	0	0	0	0	0	0	0	14,27	35,6	0	30,27	0	0	11,11	0	0	0	19,8	0	0	3,33	0	0
Mycosphaerellaceae	0	0	0	0	0	0	0	0	0	0	0	1,02	0	0	0	0	0	0	44,4	0	0	0	0	0	0	0	0	0	0	0	0	0	0	0	0
o__Capnodiales;f__NA	0	0	0	0	0	0	0	0	0	0	0	0	0	0	0	0	0	0	27,78	0	0	0	0	0	0	0	0	0	0	0	0	0	0	0	0
Didymellaceae	0	0	0	0	0	0	0	0	0	0	42,17	0	0	0	0	0	0	0	0	0	0	0	0	0	71,99	0	0	0	0	0	0	84,25	43,1	0	0
Didymosphaeriaceae	0	0	0	0	0	0	0	0	0	0	57,84	0	0	0	0	0	0	0	0	0	0	0	0	0	0	0	0	0	0	0	0	0	0	0	0
Trichomeriaceae	93,44	0	0	0	0	0	0	0	0	0	0	0	0	0	0	0	0	0	0	0	0	0	0	0	0	0	0	0	0	0	0	0	0	0	0
Aspergillaceae	0	0	0	41,34	0	0	0	0	35,05	59,88	0	0	0	0	0	0	0	0	27,82	0	0	0	18,81	0	0	0	0	0	0	0	0	15,75	18,78	0	25,78
Thermoascaceae	0	0	0	2,34	0	0	0	0	0	0	0	0	0	0	0	13,45	0	0	0	0	0	0	0	0	0	0	0	0	0	0	0	0	0	0	0
Sclerotiniaceae	0	0	0	0	0	0	0	0	0	0	0	4,84	0	0	0	0	0	0	0	0	0	0	0	0	0	0	0	0	0	0	0	0	0	0	0
Rhytismataceae	0	0	0	0	0	0	0	0	0	0	0	0,63	0	0	0	0	0	0	0	0	0	0	0	0	0	0	0	0	0	0	0	0	0	0	0
p__Ascomycota;c__NA;o__NA;f__NA	0	0	1,79	0	0	0	0	0	0	0	0	2,84	0	0	0	0	0	0	0	0	0	0	0	0	0	0	0	21,79	0	0	0	0	0	0	0
Pneumocystidaceae	0	53,15	0	19,77	0	0	0	0	56,66	0	0	0	0	0	0	4,08	0	0	0	0	0	0	50,92	0	0	0	0	0	0	80,2	62,42	0	0	0	0
Debaryomycetaceae	0	0	0	0	100	0	0	0	0	0	0	0	0	0	0	0	0	0	0	0	0	0	0	0	6,05	0	0	0	80,93	0	0	0	0	0	0
Dipodascaceae	0	0	94,13	0	0	0	0	0	0	0	0	0	0	0,02	0	0	0	0	0	0	0	0	0	0	0	0	0	0	0	0	0	0	0	0	0
Pichiaceae	0	0	0	0	0	0	0	0	0	0	0	0	0	0	0	0	0	0	0	0	18,88	0	0	0	0	0	0	0	0	0	0	0	0	0	0
Saccharomycetaceae	0	0	0	0	0	0	10,59	0	0	0	0	0	0	0	0	0	0	0	0	0	0	0	0	0	0	0	0	0	0	0	0	0	0	0	0,28
Saccharomycetales_fam_Incertae_sedis	0	0	0	0	0	0	0	0	0	0	0	0	0	0	0	0	0	0	0	0	0	100	0	0	0	0	0	53,91	19,07	0	0	0	0	0	73,94
Hypocreales_fam_Incertae_sedis	0	0	0	0	0	0	22,4	13,51	0	0	0	0	0	0	9,73	5,36	0	0	0	0	0	0	0	0	0	0	20,3	0	0	0	0	0	0	0	0
Nectriaceae	6,56	0	0	0	0	0	0	0	0	0	0	10,55	0	0	0	0	0	0	0	0	0	0	0	0	0	36,77	0	0	0	0	0	0	0	0	0
c__Sordariomycetes;o__NA;f__NA	0	0	0	0	0	0	2,7	0	0	0	0	0	0	0	0	0	0	0	0	0	0	0	0	0	0	0	0	0	0	0	0	0	0	0	0
Crepidotaceae	0	0	0	0	0	0	0	0	0	0	0	5,56	0	0	0	0	0	0	0	0	0	0	0	0	0	0	0	0	0	0	0	0	0	0	0
Stephanosporaceae	0	0	0	0	0	0	0	0	0	0	0	10,91	0	0	0	0	0	0	0	0	0	0	0	0	0	0	0	0	0	0	0	0	0	0	0
Tricholomataceae	0	0	0	0	0	49,26	0	0	0	0	0	2,69	0	0	0	0	0	0	0	0	0	0	0	0	0	45,84	0	0	0	0	0	0	0	0	0
Atheliaceae	0	0	0	0	0	0	0	11,4	0	0	0	0	0	0	0	0	0	0	0	0	0	0	0	0	0	0	0	0	0	0	0	0	0	0	0
Suillaceae	0	0	0	0	0	0	0	0	0	0	0	0	81,91	0	0	0	0	0	0	0	0	0	0	0	0	0	0	0	0	0	0	0	0	0	0
Schizoporaceae	0	0	0	0	0	0	0	0	0	0	0	21,05	0	0	0	0	0	0	0	29,9	0	0	0	0	0	0	0	0	0	0	0	0	0	0	0
c__Agaricomycetes;o__NA;f__NA	0	0	0	0	0	0	0	0	0	0	0	14,34	0	0	0	0	0	0	0	12,13	0	0	0	0	0	0	0	0	0	0	0	0	0	0	0
Meruliaceae	0	0	0	0	0	0	0	0	0	0	0	0	0	0	0	0	0	0	0	0	0	0	0	0	0	0	0	0	0	0	0	0	0	0	0
Steccherinaceae	0	0	0	0	0	0	0	0	0	0	0	8,85	0	0	0	0	0	0	0	0	0	0	0	0	0	0	0	0	0	0	0	0	0	0	0
Peniophoraceae	0	0	0	0	0	0	0	0	0	0	0	0	0	0	0	0	0	0	0	34,47	0	0	0	0	0	0	0	0	0	0	0	0	0	0	0
Stereaceae	0	0	0	0	0	0	0	0	0	0	0	0	0	0	0	0	0	0	0	0	0	0	0	0	0	6,28	0	0	0	0	0	0	0	0	0
Malasseziaceae	0	0	0	0,5	0	0	0	0	8,3	0	0	14,26	18,09	0	0	12,2	0	0	0	9,23	0	0	0	100	21,96	0	0	0	0	0	37,58	0	34,79	0	0
o__Malasseziales;f__NA	0	0	0	0	0	0	0	0	0	0	0	0	0	0	0	0	0	0	0	0	45,52	0	0	0	0	0	0	0	0	0	0	0	0	0	0
Filobasidiaceae	0	0	0	36,06	0	0	0	0	0	0	0	0	0	0	0	0	2,65	0	0	0	0	0	0	0	0	0	0	0	0	0	0	0	0	0	0
Tremellaceae	0	0	0	0	0	50,74	0	0	0	0	0	0	0	99,98	0	0	0	0	0	0	0	0	0	0	0	0	0	0	0	0	0	0	0	0	0
Trichosporonaceae	0	0	0	0	0	0	0	0	0	0	0	0	0	0	0	0	0	0	0	0	0	0	0	0	0	0	0	0	0	0	0	0	0	100	0
Wallemiaceae	0	0	0	0	0	0	0	0	0	0	0	0,88	0	0	0	0	0	0	0	0	0	0	0	0	0	0	0	0	0	0	0	0	0	0	0
k__Fungi;p__NA;c__NA;o__NA;f__NA	0	7,13	4,08	0	0	0	64,31	71,98	0	40,12	0	0	0	0	90,27	64,92	97,35	100	0	0	0	0	0	0	0	0	79,7	24,3	0	0	0	0	0	0	0
